# Sodium-glucose co-transporter 2 inhibition improves age-dependent kidney microvascular rarefaction

**DOI:** 10.1016/j.kint.2025.12.011

**Published:** 2025-12-23

**Authors:** Anastasia Paulmann, Matthew D. Cox, Tom Boewer, Hannah M. Somers, Heath Fuqua, Ryan P. Seaman, Joel H. Graber, Anchal Mahajan, Cory P. Johnson, Laura L. Beverly-Staggs, Sonia Sandhi, Heiko Schenk, Hermann Haller

**Affiliations:** 1 Mount Desert Island Biological Laboratory (MDIBL), Bar Harbor, Maine, USA; 2 Division of Nephrology and Hypertension, Hannover Medical School (MHH), Hannover, Germany; 3 Mount Desert Island Biological Laboratory (MDIBL), Comparative Genomics and Data Science Core, Bar Harbor, Maine; 4 Colby College, Waterville, Maine, USA

**Keywords:** African turquoise killifish, cell-cell communication, kidney aging, metabolic shift, microvascular rarefaction, SGLT2 inhibition

## Abstract

**Introduction:**

Aging is associated with progressive loss of kidney function and vascular structure, with and without chronic kidney disease. However, the mechanisms driving kidney vascular aging and potential therapeutic interventions remain poorly understood.

**Methods:**

African turquoise killifish (Nothobranchius furzeri), a naturally short-lived vertebrate, were used to investigate the natural course of kidney aging. We inhibited the sodium-glucose co-transporter 2 using dapagliflozin (SGLT2i) to test a potential therapeutic intervention. Histological, immunofluorescent, and 3D vascular imaging were used to evaluate glomerular, tubular, vascular and functional changes. Single nuclei transcriptomic profiling was performed on whole kidneys to identify age, sex and treatment-associated molecular signatures.

**Results:**

Aged killifish kidneys exhibited hallmark features of kidney aging, including glomerulosclerosis, tubular atrophy, and vascular rarefaction. Functional changes included increased proteinuria and altered tubular transporter function. Transcriptomic profiling revealed a metabolic shift from oxidative phosphorylation to glycolysis and upregulation of pro-inflammatory pathways. Aged vasculature displayed a marked reduction in tight junctions and cell–cell contacts. SGLT2i attenuated age-related vascular rarefaction, preserved functional capillary networks, reduced albuminuria, preserved a youthful transcriptional profile and enhanced key intercellular signaling pathways.

**Conclusions:**

Our study establishes the killifish as a translational model for investigating kidney vascular aging. SGLT2i preserves kidney microvascular structure and function, reduces proteinuria, and maintains a more youthful transcriptome. These results support a vascular-protective role of SGLT2i in mitigating age-related kidney deterioration.

The kidney is particularly susceptible to age-associated deterioration. In patients with chronic kidney disease, aging accelerates disease progression, affecting approximately 10% of adults worldwide.^1^ However, therapies addressing molecular mechanisms to prevent or delay kidney aging are limited. Kidney aging features a loss of functional nephrons, tubulointerstitial fibrosis, and microvascular rarefaction.^2^ These changes collectively increase the susceptibility of patients to other age-associated conditions, including hypertension and metabolic disease. Yet, the mechanisms underlying these changes in the kidney remain incompletely understood. Microvascular rarefaction, a reduction in the density of glomerular and peritubular capillaries, is increasingly recognized as a pivotal contributor to age-related kidney decline.^3^ Capillary loss promotes local hypoxia, disrupts metabolic homeostasis, and exacerbates fibrosis, creating a self-reinforcing loop of progressive damage. Therapeutically targeting the kidney microvasculature presents a promising approach to preserving kidney health with age.

Furthermore, modeling aging longitudinally is challenging, time intensive, and expensive. Nonvertebrate models lack key mammalian-like organ systems, including vasculature and kidneys. Traditional vertebrate models, such as mice and zebrafish, have relatively long lifespans (2–5 years), limiting their utility.^4^ *Nothobranchius furzeri*, or the African turquoise killifish (hereafter referred to as killifish), is a powerful vertebrate model organism because it lives just 4 to 6 months but harbors a vertebrate-typical organ system,^5^ including a kidney structurally similar to that of humans. Despite increasing use in aging research,^6–8^ the aging killifish renal system has not been systematically characterized, and its vasculature has not been examined.

Sodium-glucose linked transporter 2 inhibitors (SGLT2i) improve kidney-related outcomes in both diabetic and nondiabetic patients. Clinical studies have shown that SGLT2i improve cardiac and kidney function,^9^ support brain health,^10^ and enhance survival in patients with chronic diseases,^11^ mostly associated with microvascular deterioration.^12,13^ Yet, little is known about their potential anti-aging effects or the underlying molecular mechanisms. We hypothesized that killifish could facilitate insights into the aging kidney and that SGLT2i could provide a model therapeutic intervention. We used conventional methods coupled with single-nucleus RNA sequencing to resolve cell type–specific transcriptome changes during aging and applied CellChat^14^ to explore alterations in intercellular signaling. We present the first systematic characterization of killifish kidney aging, focusing on vascular rarefaction and age-related proteinuria onset. We found that aging is associated with progressive microvascular rarefaction and glomerular dysfunction. Moreover, SGLT2i treatment preserves vascular integrity and is associated with a lower incidence of proteinuria in aged fish.

## METHODS

Detailed protocols are available in the Supplementary Methods. This article was prepared in accordance with the ARRIVE (Animal Research: Reporting In Vivo Experiments) 2.0 guidelines,^15^ and the Supplementary ARRIVE reporting checklist is provided as Supplementary Material.

### Animal husbandry, lifespan analysis

Wild-type *Nothobranchius furzeri* (strain GRZ) were maintained at 27 °C on a 12-hour light/dark cycle. Water conditions were controlled (pH, 7.0–7.4; conductivity, 2800 μS). For aging studies, fish were housed in a recirculating system; for SGLT2i experiments, fish were maintained in static tanks with daily manual water changes. Adult fish were fed Artemia and Killifeast or a custom-made Repashy-based diet from 4 weeks of age (more information in Supplementary Materials_Diets). Dapagliflozin was used for SGLT2i analysis. Mating occurred weekly. Lifespan studies were performed in both (biological) sexes from 4 weeks onward. Median cohort survival defined the “old” time point.

### Histology, immunostaining, functional assays, and quantification

Fish were euthanized with 0.1% MS-222, and kidneys were fixed in 4% paraformaldehyde, paraffin, embedded, and sectioned at 10 μm. Sections were stained with hematoxylin and eosin or used for immunofluorescence (SGLT2 on sections; CD31 on whole-mount kidneys). After blocking with 10% normal goat serum, tissues were incubated with primary antibodies at 4 °C, followed by Alexa Fluor–conjugated secondary antibodies. Imaging was performed on Zeiss Axio Observer Z1 or LSM 980 with Airyscan. Whole kidney CD31^+^ area was quantified using Fiji and normalized to total kidney area. For vascular leakage assays, young and old fish were perfused intracardially with 0.1% fluorescent albumin; kidneys were fixed 48 hours later and imaged via spinning-disk confocal microscopy (CSU-W1; Yokogawa), and albumin^+^ areas were quantified as a percentage of kidney area. For *ex vivo* tubular transport, kidneys were incubated in marine teleost buffer with fluorescent methotrexate or 2-(N-(7-nitrobenz-2-oxa-1,3-diazol-4-yl)amino)-2-deoxyglucose (2-NBDG), ± multidrug resistance-associated protein 2 (MRP2) inhibitor or SGLT2i, and imaged by spinning-disk confocal microscopy; fluorescence intensity ratios (cell/lumen) were analyzed in Fiji. For 3-dimensional vascular analysis, fish were perfused with 3% gelatin containing 0.1% fluorescent albumin, and kidneys were cleared with formamide and imaged by LSM 980. Vascular volume, length, density, and branch points were quantified using Fiji, Amira, and WinFiber3D for the entire kidney.

### Single-nucleus RNA sequencing

Our full single-nuclei isolation protocol is available in the Supplementary Methods. Libraries were prepared using the 10x Genomics Chromium Single Cell 3′ v3.1 platform and sequenced on an Illumina NovaSeq 6000. The single-nucleus RNA-sequencing datasets generated during this study as well as processed data and code used for analysis are available in the Gene Expression Omnibus repository under accession number GSE297623. Additional information is available from the corresponding author on request.

### Statistical analysis

We analyzed all data in GraphPad Prism. Outliers were excluded using a Q = 1% threshold. Normality was assessed via Shapiro-Wilk test. Parametric (unpaired *t* test, 1-way analysis of variance with Tukey *post hoc*) or nonparametric (Mann-Whitney, Kruskal-Wallis) tests were used as appropriate. Each data point displayed represents a biological individual; technical replicates based on experimental conditions varied between 3 and 12. Data are shown as mean ± SD; significance was defined as *P* ≤ 0.05 (*), *P* ≤ 0.01 (**), and *P* ≤ 0.001 (***).

## RESULTS

### Killifish exhibit advanced aging with stable organ scaling

**Figure 1 |:**
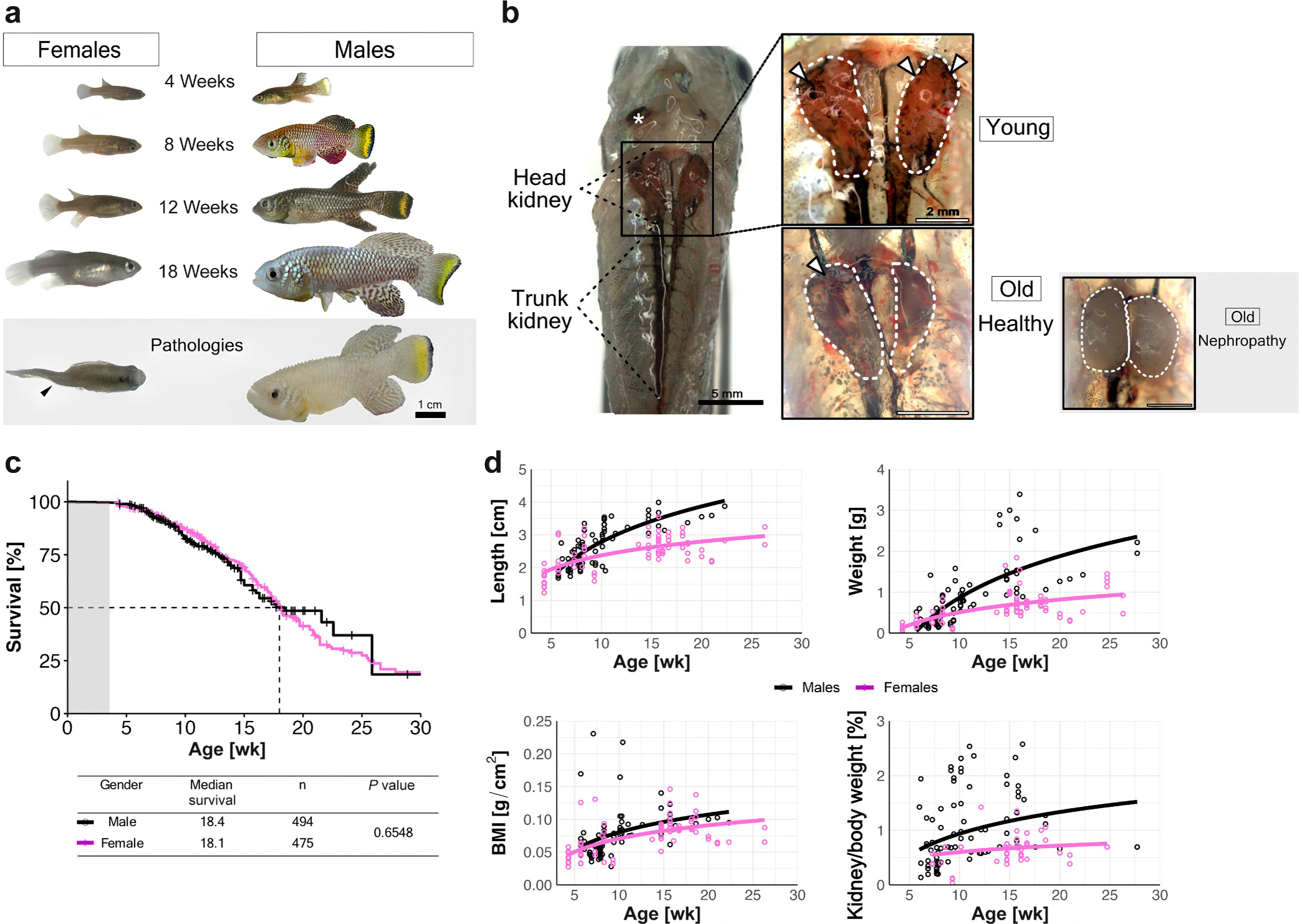
Aging-associated changes and nephropathy in African turquoise killifish. (**a**) Representative images of female and male killifish at 4, 8, 12, and 18 weeks of age. Lower panel shows phenotypic pathologies in old fish: spinal kinking (left; arrowhead indicates body deformation) and dropsy (right). Bar = 1 cm. (**b**) Representative morphology of killifish kidney anatomy (represented in young male killifish). Dotted lines show head kidney and trunk kidney structures. Insets show representative head kidneys at young and old age. The asterisk marks the corpuscle of Stannius. White arrowheads point to melanocytes covering the kidneys. Old fish show examples of healthy kidneys and nephropathy. Bars = 5 mm (left); 2 mm (right). (**c**) Survival curves of male (black) and female (magenta) killifish over time. (**d**) Growth parameters over time, including body length, body weight, body mass index (BMI), and kidney-to-body weight ratio, stratified by sex. Data points represent individual animals; lines represent fitted regression curves (n = 60 male fish; n = 50 female fish).

**Figure 2 |:**
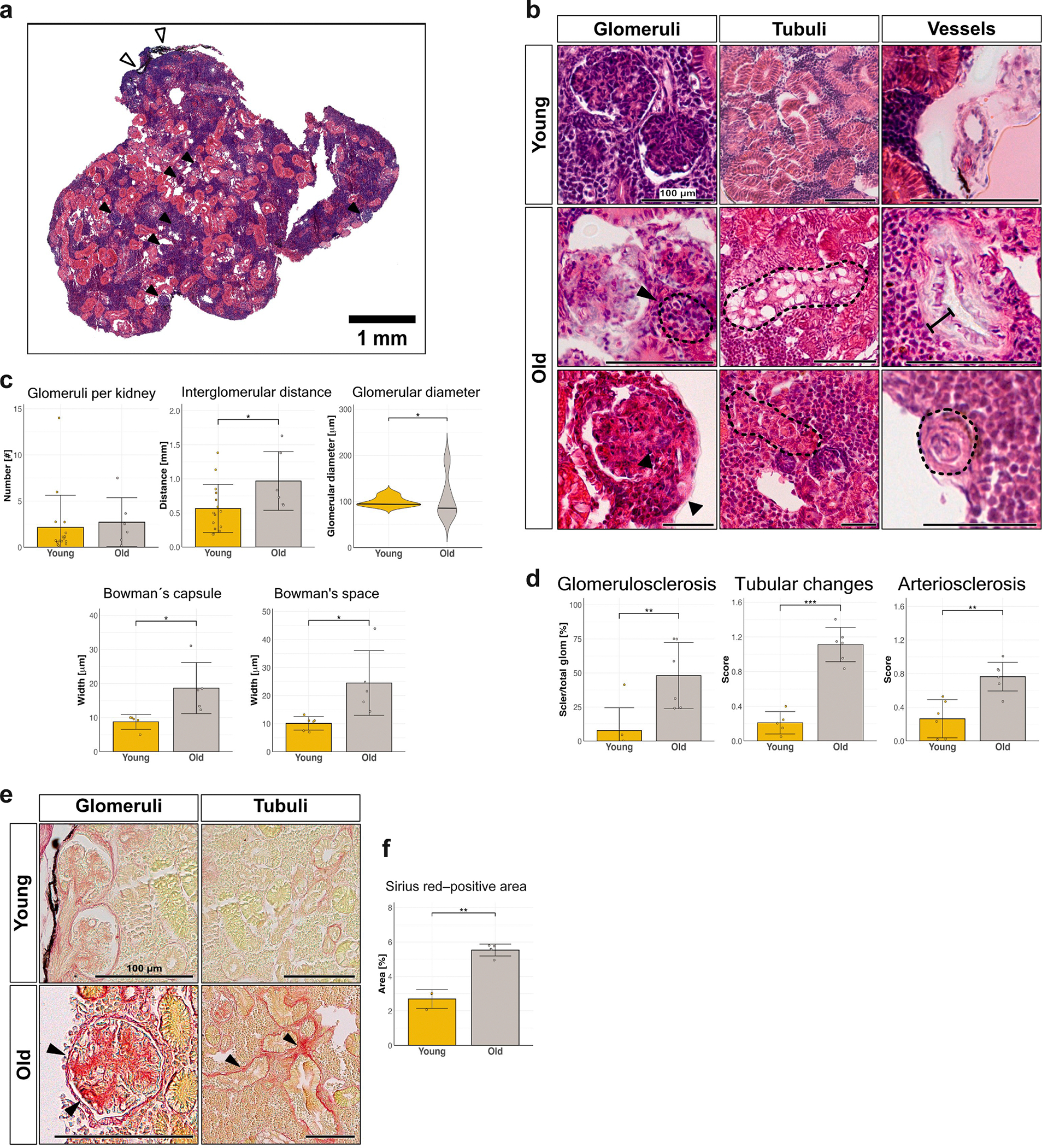
Aging killifish kidneys are characterized by nephrosclerosis and fibrosis. (**a**) Representative hematoxylin and eosin (H&E) staining of young male killifish kidney. Fish kidney does not show distinct cortical and medullary organization, as seen by evenly dispersed glomeruli (black arrows). White arrowheads show melanocytes in kidney capsule. (**b**) Representative H&E staining of glomeruli, tubuli, and vessels from young (top) and old (bottom) killifish kidneys. Dotted outlines indicate pathologic glomeruli and tubuli (e.g., sclerosed glomeruli, tubular atrophy, and vacuolized tubules). Black arrowheads point to mesangial matrix thickening. Black lines show vessel wall thickening (e.g., media hyperplasia) and intimal proliferation in vessels. Bar = 100 μm. (**c**) Morphometric analysis of glomeruli showing stable glomerular counts but increased interglomerular distance (*P* = 0.0266), suggesting a decrease in glomerular density, greater glomerular size variability with age (*P* = 0.0128 using F-test), and mesangial matrix expansion (*P* = 0.0219) and Bowman’s space expansion (*P* = 0.0146) (n = 6 to 16 young; n = 6 old). (**d**) Quantification of glomerulosclerosis (number of sclerosed to total glomeruli): 7.7% ± 17% in young, 48% ± 24% in old animals (*P* = 0.013), tubular atrophy (*P* = 0.0001), and arteriosclerosis scores (*P* = 0.0015) (n = 6 young; n = 6 old). (**d**) Representative Sirius red staining reveals increased collagen staining in glomerular and tubular regions (arrowheads). Bar = 100 μm. (**e**) Quantification of Sirius red–positive area as percentage of total tissue area (n = 3 young; n = 4 old). To optimize viewing of this image, please see the online version of this article at www.kidney-international.org.

Killifish exhibit rapid growth, early sexual maturation, and a compressed aging timeline (Figure 1a). The killifish kidney consists of 2 anatomically distinct regions (Figure 1b). The cephalad (head) kidney contains glomeruli, tubules, and hematopoietic tissue, which is regarded as the teleost bone marrow equivalent.^16^ The caudal (or trunk) kidney holds primarily collecting ducts. Both kidney regions are covered in pigmented melanocytes (arrowheads; referred to as melanomacrophages^16^), giving them a speckled appearance. On top of the cephalad kidneys lies the corpuscle of Stannius (*), a small endocrine gland responsible for calcium regulation found in the kidneys of teleost fish. Unlike in humans or mice, fish kidney lacks a distinct cortical and medullary organization, as glomeruli and tubules are evenly dispersed throughout the head kidney (Figure 2a).

In our facility, the median lifespan of the GRZ strain, which is the shortest lived killifish strain,^17^ is approximately 18 weeks (Figure 1c). Sexual maturity is reached by 4 to 6 weeks of age. Sex-specific differences in lifespan are mitigated when animals are housed separately, whereas group housing led to shorter lifespan in males than females (Supplementary Figure S1A). Killifish exhibit indeterminate growth, with both length and weight increasing—albeit at a reduced rate with age—whereas body mass index remains relatively stable (Figure 1d). Despite continuous somatic and renal growth, the kidney-to-body weight ratio remains stable, varying only approximately 0.5% to 1% across age and sex, suggesting proportional organ scaling during aging (Figure 1d).

### Aging killifish kidneys display hallmarks of nephrosclerosis

Aging in killifish kidneys is characterized by progressive structural deterioration, consistent with hallmarks of nephrosclerosis. Kidney histology reveals a marked contrast between young and aged kidneys (Figure 2b). In young kidneys, glomeruli, tubules, and vasculature maintain an organized and healthy morphology. In contrast, kidneys of old killifish exhibit an increased proportion of glomerulosclerosis, tubular atrophy, and vascular remodeling, including media thickening of larger vessels and intimal proliferations of smaller vessels.

Glomerulosclerosis is a key pathologic feature of aging in killifish. The proportion of sclerosed glomeruli is approximately 6 times greater in old animals than in young animals (Figure 2d). Mean glomerular diameter remains stable during aging, but the variance between glomerular sizes increases significantly in old kidneys (Figure 2c, panel 3). Aged kidneys also show mesangial matrix expansion with significant increase in Bowman’s space (Figure 2c, panel 4–5). Despite no change in the total glomerular count with age, the distance between individual glomeruli increases significantly in aged kidneys (Figure 2c), suggesting reduced glomerular density with age.

Aging killifish also exhibit tubular degeneration, as tubular atrophy and vacuolization become more prominent (Figure 2b), and show higher levels of tubular atrophy (Figure 2d). Arterioles exhibit pronounced age-related structural changes, including hyalinosis and thickening of the vascular walls, leading to a significant increase in arteriosclerosis (Figure 2d). Aged kidneys also show an increased fibrotic area, as measured by Sirius red staining (Figure 2e and f). Together, these findings establish the killifish kidney as a model for age-associated nephrosclerosis.

### Killifish, albuminuria, and glucose uptake

**Figure 3 |:**
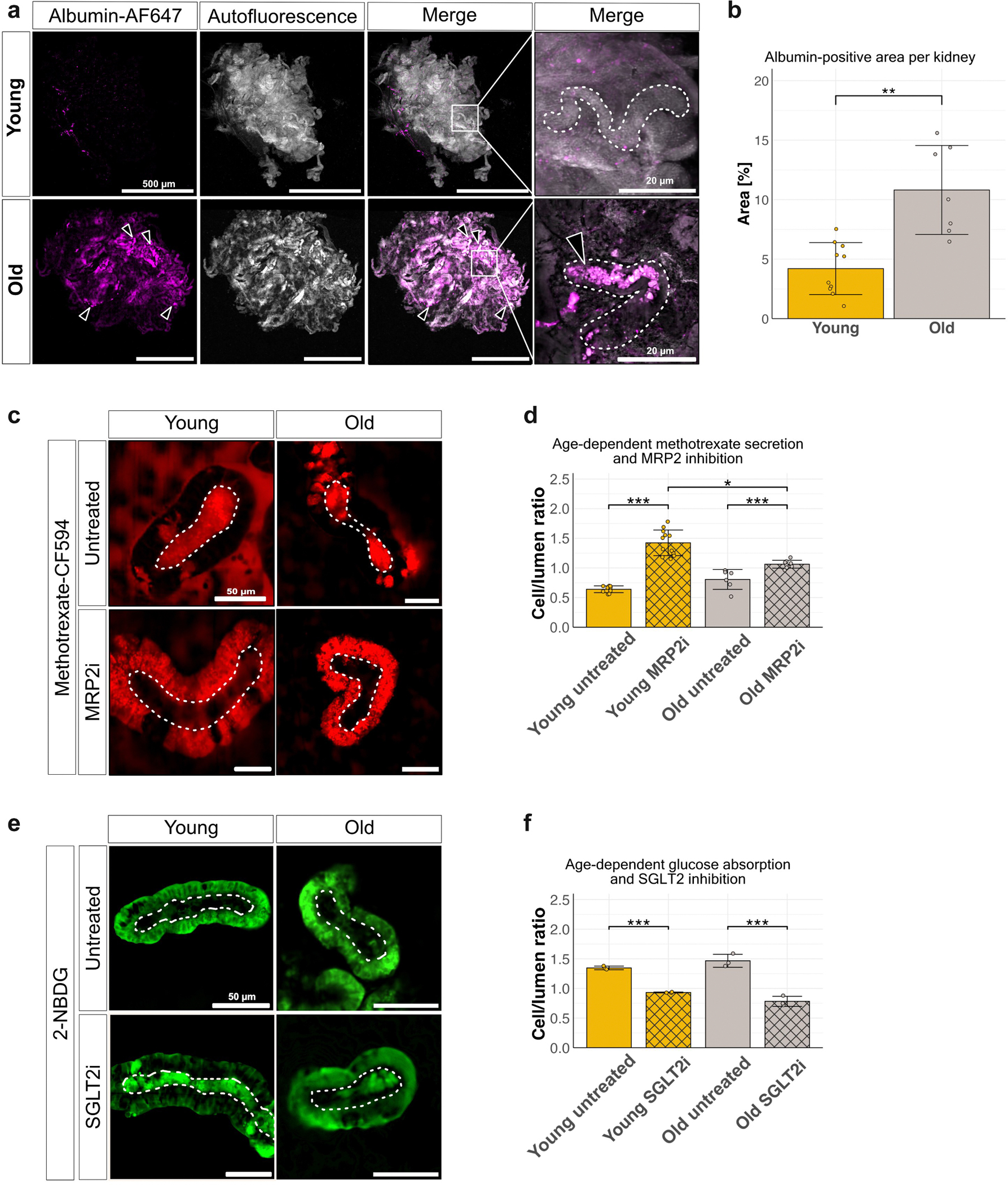
Age-dependent functional changes in the killifish kidney. (**a**) Representative images of albumin leakage after intracardial albumin injection in young (top) and old (bottom) killifish kidneys. White dotted lines outline tubules, and arrowheads point to tubules filled with albumin in aged kidneys. Bars = 500 μm; inset: 10 to 20 μm. (**b**) Quantification of albumin-positive area per kidney, showing significantly higher albumin leakage in aged kidneys (n = 10 young; n = 7 old). (**c**) Representative images of methotrexate (Mtx) secretion in untreated kidneys (top) and kidneys treated with multidrug resistance-associated protein 2 (MRP2) inhibition (bottom). Untreated tubules secrete Mtx into their lumen, whereas MRP2-inhibitor treated tubules accumulate Mtx intracellularly. (**d**) Quantification of Mtx secretion (cell/lumen ratio) shows an age-dependent decline in secretion under MRP2 inhibition (*P* < 0.01) (n = 9 young untreated; n = 12 young treated; n = 6 old untreated; n = 8 old treated). (**e**) Representative images of glucose absorption visualized by 2-(N-(7-nitrobenz-2-oxa-1,3-diazol-4-yl)amino)-2-deoxyglucose (2-NBDG) in untreated tubules (top) and treated with sodium-glucose linked transporter 2 (SGLT2) inhibition (bottom). 2-NBDG is reabsorbed into the cell: with SGLT2 inhibitor (SGLT2i), 2-NBDG accumulates in the lumen. (**f**) Quantification of glucose absorption (cell/lumen ratio), showing no significant difference between young and old tubules, nor difference in response to SGLT2 inhibition (n = 3 for all groups).

We discovered that after intravascular injection of fluorescently labeled albumin (size: 67 kDa), albumin leaked from the vascular system into tubules, suggesting dysfunctional glomerular barrier integrity. To assess this finding further, we injected albumin intravascularly into young and old killifish and examined accumulation within the kidneys 48 hours after injection. In young kidneys, minimal albumin retention within kidney tubules was observed, indicating an intact filtration barrier. In contrast, aged kidneys exhibited pronounced accumulation of albumin within tubular lumens, consistent with increased glomerular permeability and protein leakage (Figure 3a and b). These findings indicate an age-dependent decline in glomerular barrier function, manifesting as albuminuria.

A physiological change in aging human kidneys is decreased functional drug secretion. We used *ex vivo* analysis of isolated kidney tubules to assess age-dependent active drug secretion. Methotrexate, a known substrate of the adenosine triphosphate (ATP)–binding cassette transporter MRP2, was used to evaluate primary active transport. Under normal conditions, methotrexate is secreted from tubular cells into the lumen, resulting in a cell/lumen ratio < 1 (Figure 3c, top panel). Pharmacologic inhibition of MRP2 reversed this gradient, causing intracellular accumulation of methotrexate (cell/lumen ratio > 1) (Figure 3c, bottom panel). Although baseline secretion was similar in young and old tubules, the effect of MRP2 inhibition was significantly attenuated in aged samples (Figure 3d), indicating an age-related decline in ATP-dependent drug transporter activity.

In contrast, proximal tubular glucose uptake remained unaffected by aging. In *ex vivo* assays, fluorescent glucose analog 2-NBDG accumulated in tubular cells from both young and old fish, yielding a cell/lumen ratio > 1 (Figure 3e, top panel). SGLT2i reduced intracellular signal and increased luminal accumulation in both groups (Figure 3e, bottom panel), consistent with effective transporter blockage. There were no significant age-dependent differences in SGLT2 activity (Figure 3f), suggesting preserved secondary active glucose reabsorption during aging.

Together, these findings indicate that although aging compromises glomerular barrier integrity and selectively impairs ATP-dependent drug secretion, key solute reabsorption processes, such as glucose uptake, remain functionally intact in aged killifish kidneys.

### Microvascular rarefaction in killifish kidney

**Figure 4 |:**
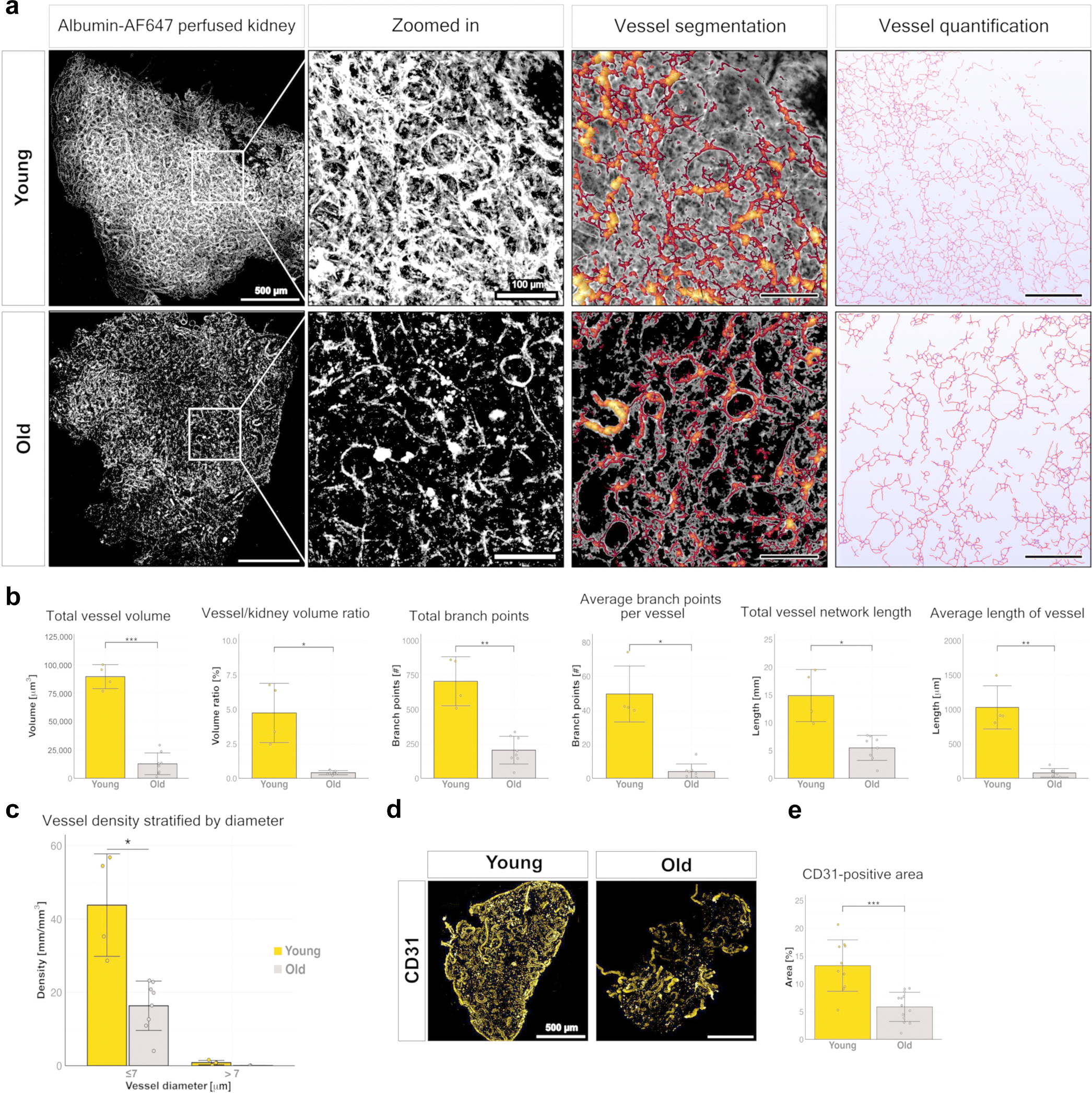
Age-associated changes in vascular morphology and density in the killifish kidney. (**a**) Representative 3-dimensional (3D) images of young (top) and old (bottom) killifish kidneys perfused with fluorescently labeled albumin hydrogel. Alterations in vascular structure are more pronounced in old kidneys. The images show a representative maximum intensity projection as well as sections with vessel segmentation performed in Amira and quantification done in WinFiber3D. Bars = 500 μm (left); 100 μm (right). (**b**) Quantification of 3D vessel morphology and volume in young (n = 4) and old (n = 8) kidneys. (**c**) Bar plot showing vessel density (mm/mm^3^) stratified by vessel diameter (≤7 vs. >7 μm) in young (yellow) and old (gray) killifish kidneys. Capillary density is significantly reduced in old kidneys (*P* = 0.0234). (**d**) Representative whole-mount immunofluorescence images of CD31 staining, in young and old kidneys. Bar = 500 μm. (**e**) Quantification of CD31-positive area relative to total kidney area (n = 12 young; n = 13 old). To optimize viewing of this image, please see the online version of this article at www.kidney-international.org.

Microvascular rarefaction—the progressive loss of glomerular and peritubular capillaries—is a defining feature of kidney aging. We assessed functional vascular density in the killifish kidney via 3-dimensional imaging of the vessel architecture using a hydrogel mixed with fluorescently labeled albumin. This method allows visualization of perfused blood vessels throughout the kidney (arterial, glomerular, and peritubular capillaries and venous branches). Young kidneys showed an extensive, highly branched vascular network, whereas aged kidneys displayed visibly reduced vascular complexity (Figure 4a). Vessel segmentation and quantification confirmed a significant decline in total vessel volume and vessel-to-kidney volume ratio with age. In addition, compared with young kidneys, aged kidneys showed a significant decrease in vascular complexity, as reflected in a significant decrease in the total number of vascular branch points (Figure 4b). Furthermore, aged kidneys showed shorter average vessel length and decreased overall network length (Figure 4b). Analysis of vessel density based on vessel diameter revealed a specific decline in capillary density—defined by vessel diameter ≤7 μm—in aged kidneys (Figure 4c), highlighting how aging affects microvasculature.

We also showed that aged kidneys exhibited a striking reduction of the vascular endothelium, as indicated by immunofluorescence staining with CD31, a pan-endothelial marker (Figure 4d). Quantification revealed that the CD31-positive area declined by >50% with age in the entire kidney (Figure 4e). This finding is consistent across sexes (Supplementary Figure S2A), although the relative reduction was more pronounced in males. These findings establish vascular rarefaction as a conserved and quantifiable hallmark of kidney aging in the killifish.

### Energy homeostasis

**Figure 5 |:**
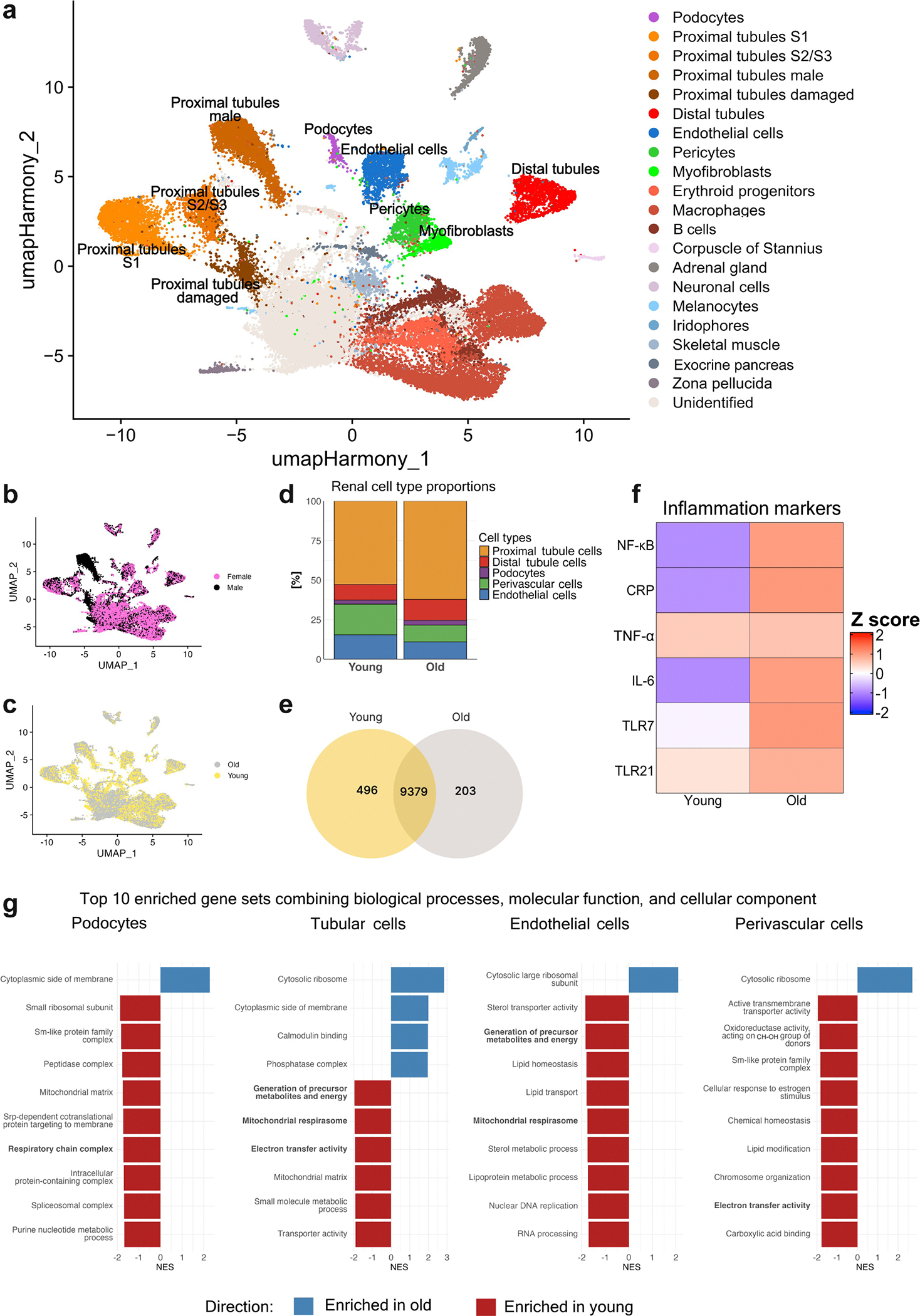
Aging alters renal cellular composition, transcriptional profiles, and energy metabolism in the killifish kidney. (**a**) Uniform manifold approximation and projection (UMAP) of single-nucleus RNA-sequencing data from young and old, male and female killifish kidneys reveals 21 distinct kidney cell clusters, annotated by cell type. (**b**) UMAP plot, colored by sex, shows substantial overlap between male and female transcriptomes, with the exception of a male-specific tubular cell cluster. (**c**) UMAP, colored by age, highlights age-associated expansion of hematopoietic populations (age-dependent cell increase by 84%) and reduced abundance of vascular cells. (**d**) Stacked bar plots showing relative proportions of major renal cell types by age. Endothelial and perivascular cells decline with age (endothelial cells decreased from 15.4% in young to 10.9% in old [−30%] and perivascular cells decreased from 19.4% to 10.7% [−45%], respectively), whereas proximal and distal tubular cell fractions increase (17% and 35%), as do podocytes (16%). (**e**) Venn diagram displaying differentially expressed genes between young and old kidneys. (**f**) Heat map of selected inflammatory markers reveals increased expression of proinflammatory genes, including *NF-κB*, *CRP*, *TNF-α*, *IL-6*, *TLR7*, and *TLR21*, in aged kidneys, supporting “inflammaging.” (**g**) Gene set enrichment analysis of aging-associated transcriptional changes in tubular cells, endothelial cells, perivascular cells, and podocytes. Top 10 enriched gene sets across combined Gene Ontology categories (biological process, molecular function, and cellular component) are shown. NES, normalized enrichment score.

We next sought to determine which cell populations show a decrease in the kidney with age. We first performed single-nucleus RNA sequencing with young and old, male and female fish, using a 10x Genomics Chromium platform. Unsupervised clustering identified 21 distinct cell populations, which were classified on the basis of their transcriptomic profile and then shown via uniform manifold approximation and projection (Figure 5a). All samples had a similar number of genes and unique molecular identifier (UMI) counts (Supplementary Figure S1B). Major kidney cell types were represented and included podocytes, 4 proximal tubule subclusters, and distal tubule cells. Cells from the collecting duct could not be confidently annotated and were excluded from further analyses. Nonepithelial populations included endothelial and perivascular cells, with the latter further subclustering into pericytes and myofibroblasts (see Supplementary Figure S3 for systemic annotations of major kidney cell types in killifish). Notably, the most abundant cell populations were derived from the hematopoietic system, comprising B cells, macrophages, and erythroid progenitor populations. In addition, several nonrenal cell types were also detected. Cell clusters lacking robust marker gene expression were omitted from downstream analyses (“unidentified”).

All major cell types were detected across age groups and sexes, with the exception of a subcluster of proximal tubule cells (proximal tubule cluster called “male”) that was predominantly male specific (Figure 5b). Despite this sex-specific distinction, proximal-tubule clusters remained spatially close to each other on the uniform manifold approximation and projection, whereas distal-tubule populations were clearly segregated. Although the overall clustering structure remained conserved across the 2 age groups (Figure 5c), quantitative analysis demonstrated a prominent expansion of hematopoietic populations with age (increase by 84%). Within kidney-resident cell types, opposing trends were observed: tubular epithelial cells and podocytes increased in proportion, whereas the proportion of vascular-associated cell types (i.e., endothelial cells [ECs] and perivascular cells) declined (Figure 5d). These results suggest that aging in the killifish kidney is accompanied by a shift toward epithelial cell predominance at the expense of vascular and stromal cell populations, consistent with microvascular rarefaction observed in histologic and 3-dimensional imaging analyses. Differential gene expression analysis revealed substantial transcriptional remodeling with age. Of the 10,078 genes detected across conditions, 496 were unique to young kidneys and 203 were unique to old kidneys (Figure 5e). In addition, a heat map of canonical inflammatory markers showed upregulation of proinflammatory genes in aged kidneys, consistent with a transcriptional signature of chronic low-grade inflammation (“inflammaging”; Figure 5f).

Gene set enrichment analysis across 4 major kidney cell types revealed consistent downregulation of mitochondrial and energy-related pathways with age (Figure 5g), supporting an age-associated metabolic shift away from oxidative phosphorylation toward less efficient energy production pathways. Endothelial and perivascular cells showed widespread suppression of lipid transport, sterol metabolism, and oxidoreductase activity, indicating a decline in both metabolic and biosynthetic functions. In ECs, a metabolic shift toward glycolysis can be observed with negative enrichment of the mitochondrial energy pathway as well as pathways related to lipolysis and amino acid metabolism. In podocytes, negative enrichment was observed for mitochondrial matrix– and respiratory chain–related gene sets. Perivascular cells displayed reduced activity in chromatin and chemical homeostasis pathways (see Supplementary Figure S4 for an in-depth overview of the gene set enrichment analysis results). Together, these findings reveal that aging in the killifish kidney is characterized by loss of mitochondrial energy production and a metabolic shift, particularly affecting vascular compartments, and strongly correlates with structural and functional renal decline.

### Age, endothelial cell-cell contacts, and cellular communication

**Figure 6 |:**
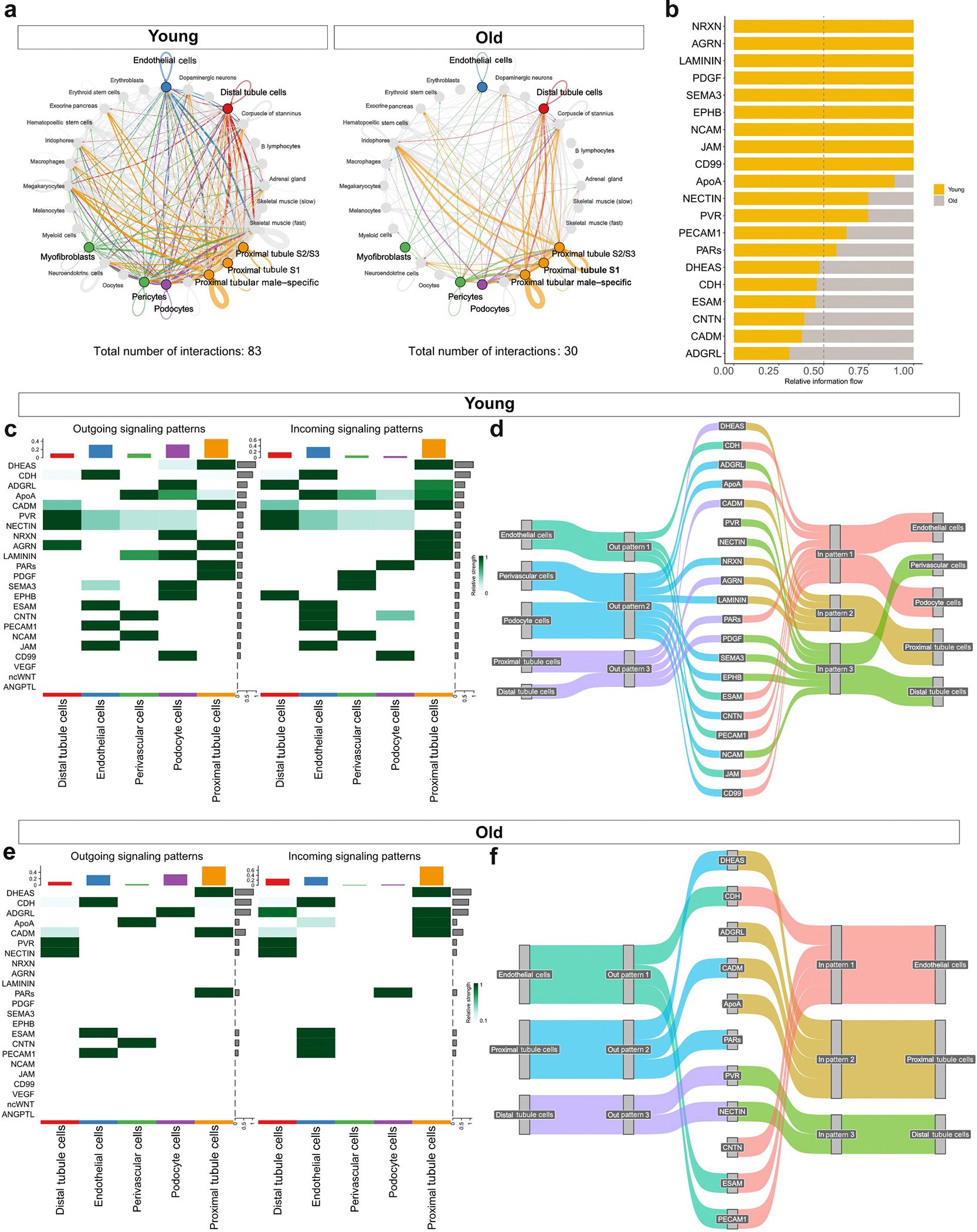
Aging disrupts cell-cell communication networks in the killifish kidney. CellChat analysis was performed on single-nucleus RNA-sequencing data from kidneys of young and old killifish. (**a**) Circle plots visualize global intercellular communication networks in young (left) and old (right) kidneys. Each node represents a cell type, and each connecting line represents a significant interaction, with width proportional to communication strength. Cell types of interest are colored, and nonrenal cell types are displayed in gray. The total number of interactions decreases from 83 in young to 30 in old kidneys. (**b**) Bar graph shows relative information flow of individual signaling pathways in young (yellow) and old (gray) kidney cells, highlighting selective retention or loss of pathway activity with age. (**c,e**) Heat maps show outgoing (left) and incoming (right) signaling patterns in (**c**) young and (**e**) old kidneys. Pathways are sorted by signaling in young kidneys. Aging reduces both the diversity and magnitude of signaling activities across cell types. (**d,f**) Sankey plots illustrate dominant outgoing signaling pathways in (**d**) young and (**f**) old kidney cells, demonstrating diminished pathway complexity and redistribution of signaling roles with age. ADGRL, adhesion G protein–coupled receptor L (latrophilin); AGRN, agrin; ANGPTL, angiopoietin-like protein; ApoA, apolipoprotein A; CADM, cell adhesion molecule; CDH, cadherin; CNTN, contactin; DHEAS, dehydroepiandrosterone sulfate; EPHB, ephrin type-B receptor; ESAM, endothelial cell-selective adhesion molecule; JAM, junctional adhesion molecule; NCAM, neural cell adhesion molecule; ncWNT, noncanonical Wingless-related Int-1; NRXN, neurexin; PAR, protease-activated receptor; PDGF, platelet-derived growth factor; PECAM, platelet–endothelial cell adhesion molecule; PVR, poliovirus receptor; SEMA, semaphorin; VEGF, vascular endothelial growth factor.

We next investigated age-related changes in intercellular communication in the killifish kidney using CellChat to infer ligand-receptor interactions between kidney and vascular cell types. Overall, the total number of ligand-receptor interactions declined with age across all kidney cell types (Figure 6a), reflecting reductions in cell-cell adhesion, secreted signaling molecules, and extracellular matrix components (Figure 6b).

A heat map of signaling patterns in young and old kidneys shows marked loss of intercellular communication with age (Figure 6c and e). Compared with young kidneys, old kidneys show substantially reduced EC autocrine signaling, as junctional adhesion molecules, nectins, and poliovirus receptor are lost in old kidneys. Furthermore, old kidneys exhibit reduced signaling of perivascular cells to ECs via contactin (CNTN) and apolipoprotein A and diminished perivascular extracellular matrix signaling, as evidenced by reduced laminin, nectin, and poliovirus receptor signaling. Perivascular cells and podocytes show the strongest age-associated loss of signaling pathways, suggesting an impaired ability of podocytes and perivascular cells to maintain extracellular matrix and basement membrane. Together, these alterations indicate a profound disruption of vascular and extracellular homeostasis with aging.

### SGLT2 preserves kidney vessels and reduces proteinuria

**Figure 7 |:**
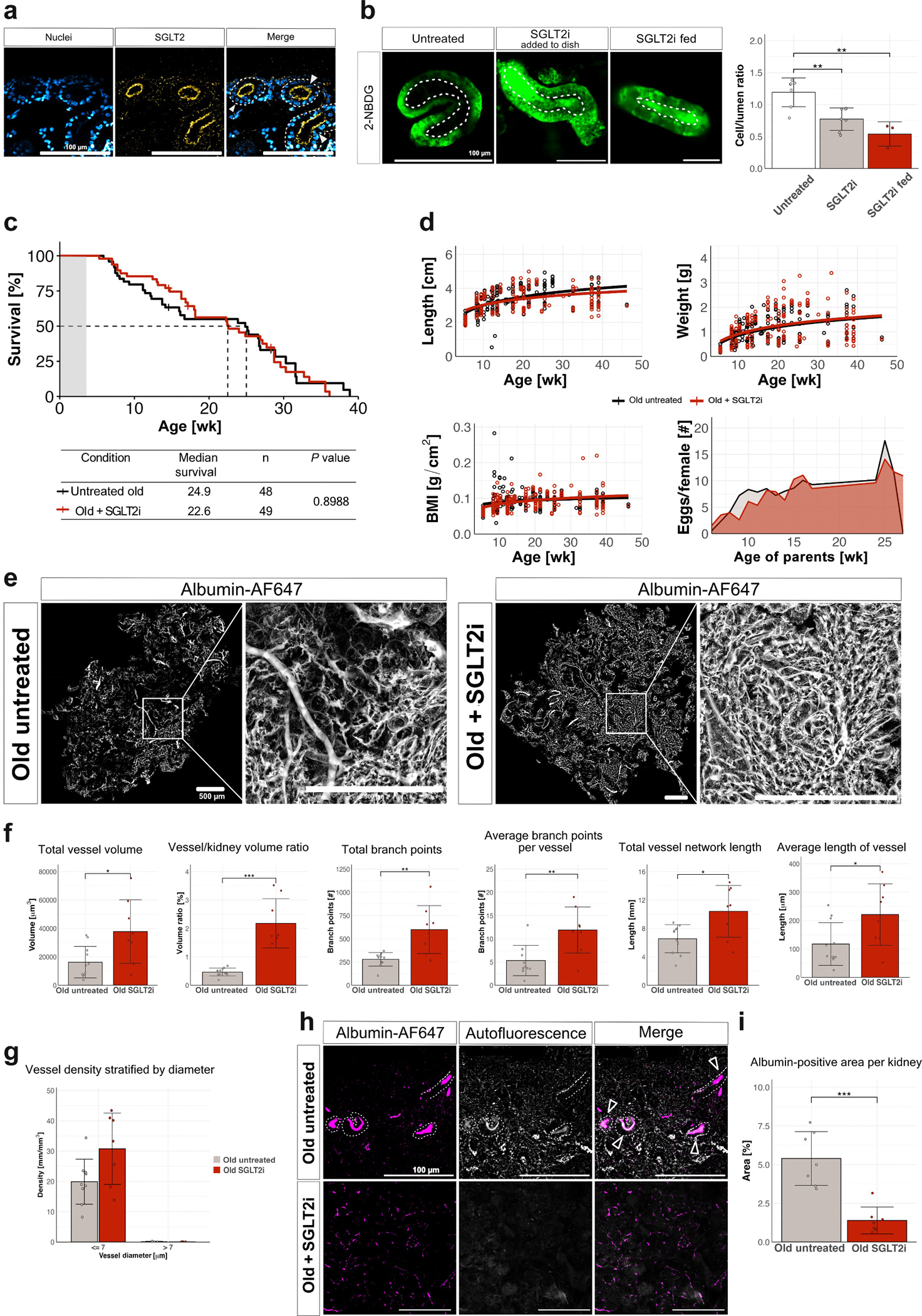
Sodium-glucose linked transporter 2 (SGLT2) inhibition preserves kidney vasculature and reduces proteinuria in aging killifish without extending lifespan. (**a**) Representative images of kidney section from old untreated killifish stained for SGLT2 (yellow) using a custom-made antibody and nuclei (blue). SGLT2 expression is observed on the luminal side of tubules. Bar = 200 μm. (**b**) Representative image of glucose absorption, visualized by the fluorescent glucose analog 2-(N-(7-nitrobenz-2-oxa-1,3-diazol-4-yl)amino)-2-deoxyglucose (2-NBDG) (green), showing decreased glucose uptake in the tubules of old fish kidneys fed with SGLT2 inhibitor (SGLT2i). Quantification of the cell/lumen ratio shows a significant decrease in glucose uptake in old SGLT2i-treated kidneys compared with untreated controls, and no difference between tubules treated directly with SGLT2i *ex vivo* (gray bar) and fish fed SGLT2i (red bar) (n = 7 untreated; n = 7 SGLT2i added directly to tubules; n = 3 SGLT2i fed). (**c**) Survival curves comparing untreated and SGLT2i-treated killifish, with median survival at 24.9 weeks for untreated fish and 22.6 weeks for SGLT2i-treated fish. Statistical analysis indicates no significant difference in survival between groups (*P* = 0.8988). (**d**) Growth parameters of untreated and SGLT2i-treated killifish, including body length, weight, body mass index (BMI), and fecundity (number of eggs per female). SGLT2 inhibition has minimal to no effect on growth, weight, or reproductive parameters. (**e**) Representative images of vessel visualization in old untreated and old SGLT2i-treated kidneys. Kidneys were perfused with albumin. Vessel networks are segmented and quantified, showing differences in vascular structure. Bar = 500 μm. (**f**) Quantification of 3-dimensional vessel morphology and volume in old untreated and old SGLT2i-treated kidneys. Total vessel volume, vessel-to-kidney volume ratio, and total branch points are significantly higher in the SGLT2i-treated group. Other vessel parameters, such as average branch points per vessel, vessel network length, and average vessel length, also show significant improvements with treatment. Bar plot shows a trend in restoring vessel density (mm/mm^3^) of vessels with diameter ≤7 μm in untreated (gray) and SGLT2i-treated (red) killifish kidneys (*P* = 0.0548), with no difference in vessels with diameter >7 μm (n = 11 untreated; n = 8 SGLT2i treated). (**g**) Bar plot showing vessel density (mm/mm^3^) stratified by vessel diameter (≤7 vs. >7 μm) in old untreated (gray) and old SGLT2i-treated (red) killifish kidneys. Capillary density is increased in SGLT2i-treated kidneys in a sex-specific manner; however, the combined analysis does not show significance (*P* = 0.1; see Supplementary Figure S1F). (**h**) Representative images of albumin leakage in old untreated and old SGLT2i-treated kidneys. Arrowheads indicate albumin leaked into tubules. SGLT2i treatment reduces albumin leakage in the tubules of aged kidneys. Bar = 100 μm. (**i**) Quantification of albumin-positive area per kidney (n = 7 per group). To optimize viewing of this image, please see the online version of this article at www.kidney-international.org.

SGLT2 inhibition is an established route to maintain kidney health. To evaluate its role in the killifish kidney, we first confirmed the presence of SGLT2 via immunostaining, which revealed apical localization of SGLT2 in proximal tubular cells in aged kidneys (Figure 7a). We used the SGLT2 inhibitor dapagliflozin in our treatment experiments. To administer dapagliflozin, we developed a custom diet incorporating the drug, thereby enabling long-term pharmacologic inhibition while avoiding the substantial problems with water-based delivery (e.g., hydrolysis of dapagliflozin, high drug requirements, uncertain absorption through the gills, and unknown accumulation dynamics). Functional inhibition was verified using an *ex vivo* tubule assay: fish fed the SGLT2i-enriched diet exhibited significantly reduced uptake of the fluorescent glucose analog 2-NBDG compared with fish fed a regular diet (Figure 7b). The cell-to-lumen ratio was comparable between fish that were fed the drug and tubules treated directly with SGLT2i *ex vivo* (Figure 7b), confirming effective systemic delivery and target engagement.

We found that although tubules treated directly with dapagliflozin *ex vivo* robustly inhibited glucose uptake (Figure 7b), dapagliflozin treatment did not extend lifespan in the GRZ strain (Figure 7c). Sex-specific analysis showed a trend of extended longevity in female fish, but no difference in males (Supplementary Figure S2). SGLT2i treatment resulted in no significant changes in body length, weight, body mass index, or fecundity (Figure 7d).

Nonetheless, SGLT2 inhibition preserved kidney vascular structure in aged animals, as indicated by 3-dimensional reconstruction of perfused dapagliflozin-treated kidneys and untreated age-matched controls (Figure 7e). Quantitative analysis confirmed that SGLT2i resulted in significant increases in total vessel volume, vessel-to-kidney volume ratio, and total branch points (Figure 7f). However, these effects were far more pronounced in female fish than in males (Supplementary Figure S2C). Capillary density showed a trend toward recovery with SGLT2i treatment, albeit not statistically significant (Figure 7g). Significance was reached, however, in female kidneys, but not in males (Supplementary Figure S1F).

SGLT2 treatment also partially restored glomerular filtration barrier function. *In vivo* albumin leakage assays revealed a reduction in albumin-positive tubules in SGLT2i-treated kidneys (Figure 7h), and quantification confirmed a significant decrease in albumin-positive area per kidney (Figure 7i), suggesting reduced proteinuria in SGLT2i-treated animals. This finding occurred in both sexes (Supplementary Figure S2D). In addition, treatment of old kidneys with SGLT2i resulted in an increase in CD31-positive area to almost young-like levels (Supplementary Figure S1C). Together, these findings highlight the potential of SGLT2i to improve kidney health independently of lifespan extension.

### Mitochondrial function is improved, and inflammation is reduced

**Figure 8 |:**
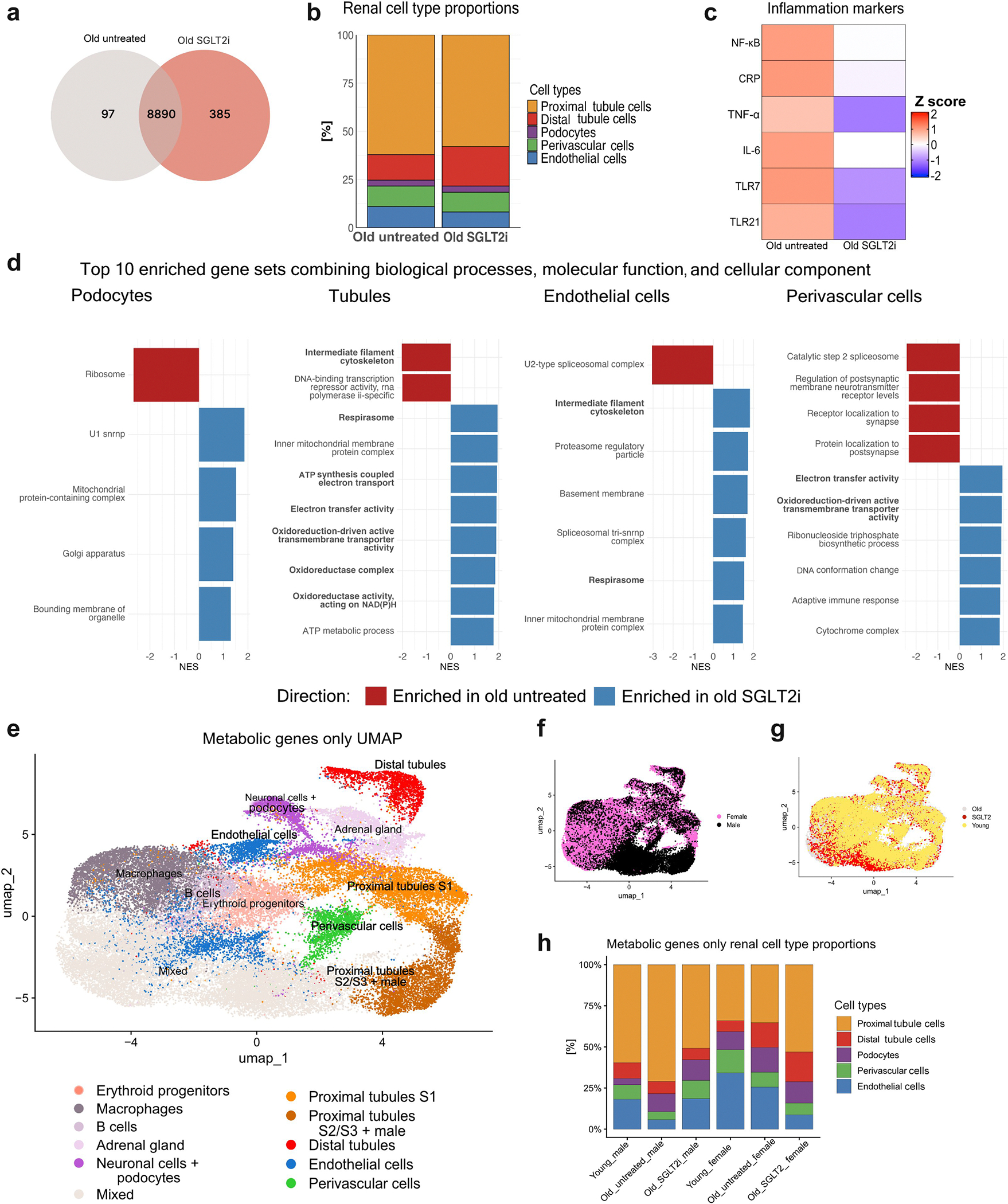
Sodium-glucose linked transporter 2 (SGLT2) inhibition restores mitochondrial function and reduces inflammatory signaling in aged killifish kidneys. (**a**) Venn diagram depicting shared and condition-specific expressed genes: 385 genes were uniquely detected in SGLT2 inhibitor (SGLT2i)–treated aged samples, whereas 97 were unique to untreated aged samples. (**b**) Bar plot showing relative proportions of major kidney cell types. No major differences were observed in endothelial and perivascular cells counts between the SGLT2i group and the untreated group. (**c**) Heat map displaying expression (Z-score) of key inflammatory genes, including *NF-κB*, *CRP*, *TNF-α*, and *IL-6*, which were downregulated in treated kidneys. (**d**) Gene set enrichment analysis of major kidney cell types showing normalized enrichment scores (NESs) for the top 10 gene sets combining biological processes, molecular functions, and cellular components. Positive enrichment (blue) highlights restored expression of mitochondrial- and electron transport–associated pathways in SGLT2i-treated animals, particularly in tubular, endothelial, and perivascular cells. (**e**) Uniform manifold approximation and projection (UMAP) visualization of single renal and nonrenal cell types from all conditions (young, old untreated, and old SGLT2i) based solely on metabolic gene expression. (**f**) Same UMAP, colored by sex, highlights a distinct male-specific proximal tubular cluster as well as differences in proximal tubule S2/S3 cluster. (**g**) UMAP, colored by age, shows largely overlapping metabolic profiles across age groups. (**h**) Stacked bar plots showing relative proportions of major renal cell types (proximal tubule, distal tubule, podocyte, perivascular, and endothelial cells) stratified by age, sex, and treatment. SGLT2 inhibition alters vascular cell proportions in males but not females.

We then tested whether treatment could reverse age-associated kidney transcriptional changes, by performing single-nucleus RNA sequencing on SGLT2i-treated killifish. Results showed that major kidney cell identities were conserved between untreated and SGLT2i-treated animals (Figure 5a). Differential expression analysis showed that although most detected genes were shared, 385 genes were unique to the SGLT2i-treated group, compared with 97 genes in the untreated aged group (Figure 8a), suggesting a treatment-induced transcriptomic shift. Proportions of vascular cell types did not differ substantially between untreated and SGLT2i-treated kidneys (Figure 8b), whereas proportions of distal tubule cells increased. Notably, expression of proinflammatory markers was attenuated in SGLT2i-treated kidneys (Figure 8c), indicative of reduced inflammaging.

Gene set enrichment analysis revealed a striking SGLT2i-mediated restoration of mitochondrial function and cellular energetics across multiple renal compartments (Figure 8d and Supplementary Figure S5). In endothelial, perivascular, podocyte, and tubular cells, SGLT2 inhibition upregulated pathways associated with electron transport activity, ATP metabolic processes, and mitochondrial membrane integrity. This reversal of age-related suppression in mitochondrial gene sets supports a treatment-induced reestablishment of oxidative phosphorylation and energy homeostasis. In tubular cells specifically, gene sets related to cytoskeletal maintenance and ATP synthesis–coupled electron transport were significantly enriched, suggesting improved structural and metabolic stability.

### Age-dependent metabolic shifts under SGLT2 inhibition

To further characterize the metabolic landscape across renal compartments in our killifish model, we leveraged an earlier approach.^18,19^ Hereby, only a subset of metabolically associated genes was retained for analysis (976 killifish-specific metabolic genes). Dimensional reduction based on metabolic genes alone preserved most major kidney cell identities, confirming compartment-specific metabolic programs (Figure 8e). Smaller nonrenal populations clustered together and were summarized under a “mixed” identity. The metabolic gene expression uniform manifold approximation and projection retained the sex-associated segregation within proximal tubule clusters (Figure 8f) but showed no major differences in overall cell type composition between aged and SGLT2i-treated samples (Figure 8g and h).

To further delineate metabolic changes, we assessed canonical pathway activity using module scores derived from representative metabolic gene sets. Across renal compartments, sex emerged as the dominant variable shaping metabolic profiles, exceeding age- or treatment-related effects (Figure 8g and Supplementary Figure S6A). Polyol, glutamine, glycolytic, and gluconeogenic scores displayed greater variability between males and females, particularly in proximal tubules and ECs. Notably, SGLT2 inhibition in males restored endothelial metabolic profiles toward a youthful state, reflected by reduced glutamine and polyol activity and normalization of glycolytic balance (Supplementary Figure S6A).

At the individual gene level, ECs demonstrated SGLT2i-mediated reversal of several age-related changes in metabolic gene expression (Supplementary Figure S6B and C), notably *AK1* (adenylate kinase 1; catalyzing adenosine diphosphate–ATP conversion), *DKC1* (dyskerin pseudouridine synthase 1; stabilizing telomerase RNA), and *PRMT1* (protein arginine methyltransferase 1; involved in endothelial nitric oxide synthase expression, nitric oxide production, and histone methylation). Collectively, metabolic gene expression analysis recapitulates previous findings, underscoring that both aging and its modulation by SGLT2 inhibition are strongly linked to metabolic remodeling within the kidney.

### SGLT2, signaling networks, and endothelial communication

**Figure 9 |:**
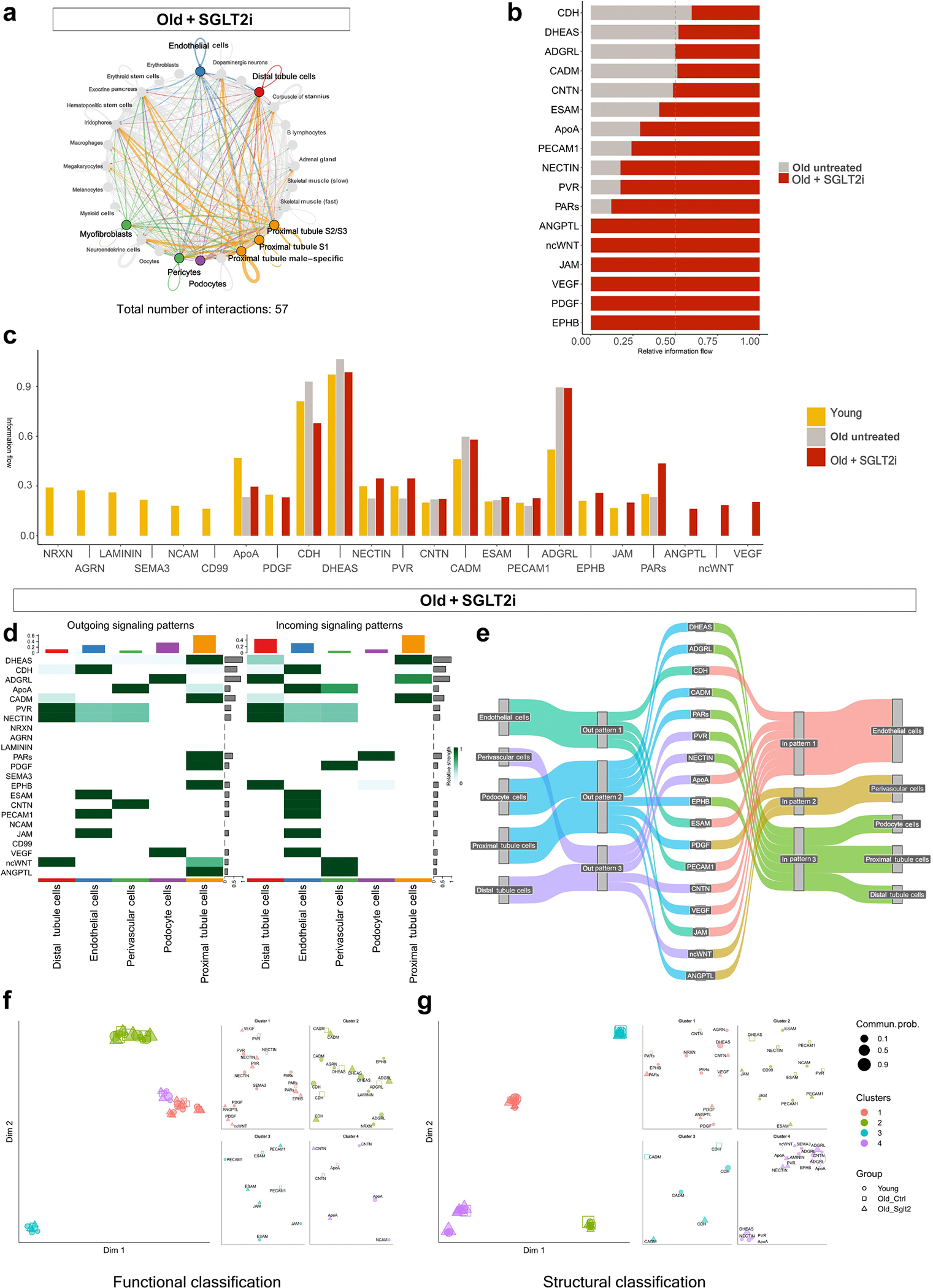
Sodium-glucose linked transporter 2 (SGLT2) inhibition restores age-related loss of cell-cell communication in the aging killifish kidney. Cell-cell signaling networks inferred using CellChat from single-nucleus RNA-sequencing data. (**a**) Circle plot depicts the global intercellular signaling network in kidneys from old fish treated with SGLT2 inhibitor (SGLT2i). Each node represents a cell type, and lines represent significant ligand-receptor interactions. The total number of interactions increased to 57 with treatment, compared with 30 in untreated old controls. (**b**) Bar plot shows relative information flow of key signaling pathways in old control (gray) versus SGLT2i-treated (red) kidneys, indicating selective reactivation of endothelial and perivascular signaling axes. (**c**) Comparison across all 3 groups (young, old control, and old + SGLT2i) highlights partial recovery of specific pathways (e.g., platelet–endothelial cell adhesion molecule 1 [PECAM1], junctional adhesion molecule [JAM], and vascular endothelial growth factor [VEGF]) toward a youthful signaling profile. Overall, SGLT2i-treated signaling pathways mimic young signaling pathways more than old untreated. (**d**) Heat maps illustrate outgoing (left) and incoming (right) signaling patterns in kidney cell types from old SGLT2i-treated kidneys. Several signaling axes are reestablished, particularly from endothelial and perivascular cells. (**e**) Sankey plot visualizes major signaling pathways restored in SGLT2i-treated kidneys, with enhanced directional signaling among glomerular and tubular compartments. (**f,g**) Joint manifold learning was used to classify signaling pathways based on (**f**) functional and (**g**) structural similarity across groups. Each dot represents a signaling pathway, clustered by similarity in sender-receiver relationships. SGLT2i-treated kidneys (triangles) show a partial shift toward the youthful communication profile (circles), distinct from untreated aged controls (squares). ADGRL, adhesion G protein–coupled receptor L (latrophilin); AGRN, agrin; ANGPTL, angiopoietin-like proteins; ApoA, apolipoprotein A; CADM, cell adhesion molecule; CDH, cadherin; CNTN, contactin; DHEAS, dehydroepiandrosterone sulfate; EPHB, ephrin-B receptor; ESAM, endothelial cell-selective adhesion molecule; NCAM, neural cell adhesion molecule; ncWNT, noncanonical Wingless-related Int-1; NRXN, neurexin; PAR, protease-activated receptor; PDGF, platelet-derived growth factor; PVR, poliovirus receptor; SEMA, semaphorin.

We also assessed the impact of SGLT2 inhibition on intercellular communication, by performing ligand-receptor network analysis using CellChat (Figure 9). The numbers of inferred interactions in untreated aged kidneys, which were lower than the numbers in young kidneys, were significantly increased following dapagliflozin treatment (Figure 9b), particularly among endothelial, perivascular, and podocyte clusters (Figure 9c). Quantitative interaction strength analysis revealed that SGLT2i preserved key vascular signaling pathways (Figure 9d). Notably, podocyte-to-endothelium paracrine signaling was enhanced under treatment, characterized by increased vascular endothelial growth factor (VEGF) A–VEGF receptor 2 signaling interaction as well as upregulation of laminin, a component of the extracellular matrix.

Outgoing and incoming signaling patterns confirmed that treated animals maintained robust vascular crosstalk (Figure 9f). In contrast to untreated aged kidneys, which exhibited collapsed or diminished vascular communication, SGLT2i preserved signaling hubs in endothelial and perivascular compartments. Dimensionality reduction of signaling networks showed that the SGLT2i-treated group clustered more closely with the youthful signature in both functional (e.g., CD99, CNTN, and VEGF) and structural (e.g., integrins, cadherins) categories (Figure 9g and h), highlighting a partial reversion to a regenerative and communicative vascular state.

In addition to preserving age-sensitive vascular signaling pathways, such as platelet-derived growth factor (PDGF), ephrin-B receptor (EPHB), and junctional adhesion molecule (JAM), SGLT2 inhibition uniquely activated a subset of signaling networks not restored in untreated aging kidneys, including angiopoietin-like proteins (ANGPTLs), noncanonical Wingless-related Int-1 (WNT), and VEGF. This pattern suggests that SGLT2i does not merely reverse age-related loss of intercellular communication but actively reconfigures the signaling landscape. The emergence of distinct ligand-receptor interactions under treatment points to a drug-specific remodeling effect, highlighting that SGLT2i exerts both rejuvenating and uniquely adaptive influences on kidney cell-cell communication during aging.

Together, these results demonstrate that SGLT2 inhibition in the aging killifish kidney restores mitochondrial bioenergetics, reduces inflammatory tone, and preserves endothelial-perivascular communication to a “youthful” state. These effects likely underlie the observed maintenance of vascular integrity despite mild reductions in EC numbers, offering a mechanistic basis for the vascular-protective effects of SGLT2 inhibition during kidney aging.

### Age-dependent remodeling of ECs and SGLT2 inhibition

**Figure 10 |:**
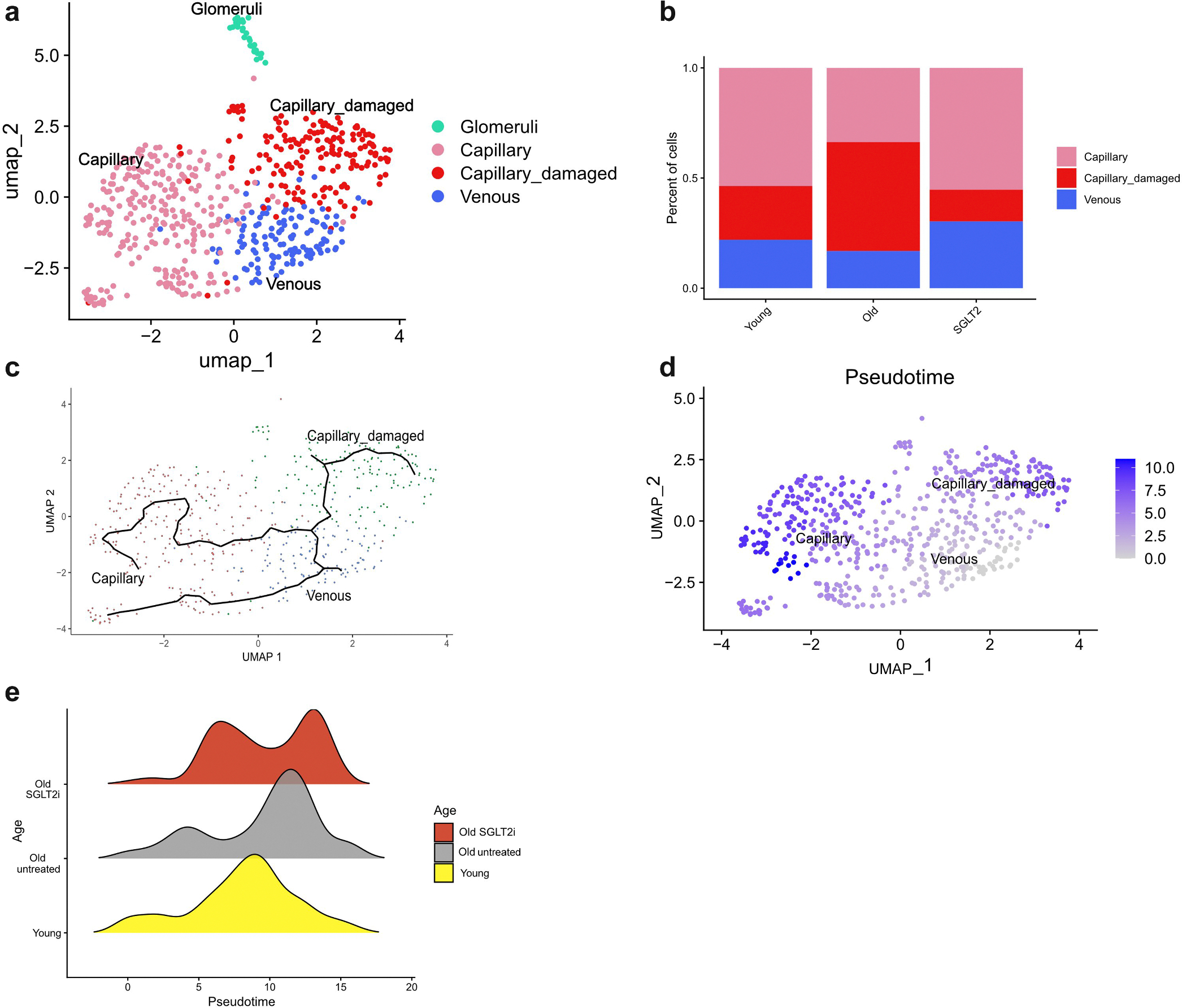
Trajectory and pseudotime analysis of kidney endothelial cells. (**a**) Uniform manifold approximation and projection (UMAP) visualization of endothelial cell subclusters integrated across all conditions. (**b**) Relative abundance of each endothelial subtype in young, old untreated, and old sodium-glucose linked transporter 2 inhibitor (SGLT2i) endothelial cells. Glomeruli endothelial cells excluded from analysis. The proportion of capillary_damaged cells increased with age, whereas SGLT2 inhibition partially restored a young-like composition. (**c**) Trajectory analysis using Monocle3 shows a branching structure connecting venous to capillary and capillary_damaged states. (**d**) Pseudotime gradient overlaid on the UMAP indicates early pseudotime in venous and capillary cells, progressing toward both intact and damaged capillary trajectories. (**e**) Density plots of pseudotime distribution by condition. Endothelial cells from old fish occupy later pseudotime states, whereas SGLT2-inhibited cells show a bimodal pattern, suggesting partial maintenance of a youthful transcriptional state as well as higher pseudotime capillary states.

To further characterize endothelial heterogeneity across age and treatment, we performed single-nucleus RNA-sequencing–based subclustering of only ECs. Four transcriptionally distinct populations were identified (Figure 10a): a glomerular cluster enriched for *KIRREL3* (also known as *NEPH2*) expression and 3 peritubular capillary subtypes comprising capillary, venous, and capillary-damaged states. Because glomerular ECs were only detected in a subset of female samples (Supplementary Figure S7A), they were excluded from downstream analyses. The capillary cluster expressed canonical endothelial genes (*CDH5*, *KDRL*), the venous cluster showed enrichment of postcapillary and inflammatory markers (*SELE*, *NR2F2*), and the capillary-damaged population exhibited intermediate features with increased ribosomal gene expression, consistent with cellular stress and cytoskeletal remodeling (Supplementary Figure S7B). Quantitatively, capillary_damaged ECs predominated in old kidneys, whereas the SGLT2i group displayed a distribution resembling young ECs (Figure 10b).

Trajectory analysis revealed a bifurcating pseudotime path originating from venous-like ECs and diverging toward either capillary or capillary-damaged fates (Figure 10c and d). Pseudotime ranking was highest in old ECs and lowest in young, with SGLT2-treated ECs showing a bimodal distribution—one fraction aligning with young-like early states, and another extending into late pseudotime (Figure 10e). This pattern suggests that SGLT2 inhibition maintains a subset of ECs in a stable, low pseudotime state compatible with preserved microvascular integrity. Among the most pseudotime-associated genes were *GATM* (creatine biosynthesis and nitric oxide synthetase pathway), which increased with age, and *CDH4*, *LAMC3*, *MAN1C1*, and *PDGFRB,* which declined, reflecting progressive loss of junctional stability, extracellular matrix organization, and pericyte signaling (Supplementary Figure S7C). These changes remain reproducible in the metabolic gene analysis only, recapitulating the 2 distinct trajectories as well as PRMT1, part of the nitric oxide synthetase pathway, as aging driver (Supplementary Figure S7D).

## DISCUSSION

We reasoned that killifish are a powerful vertebrate model to study aging across multiple organ systems, including neurodegeneration, immune senescence, and impaired regeneration. We investigated this novel animal model, established the structural, functional, and transcriptomic changes in killifish aging kidney, and evaluated the effects of SGLT2i as a potential intervention. We used state-of-the-art technologies and defined nephrosclerosis, progressive microvascular loss, age-associated albuminuria, a metabolic shift from mitochondrial respiration to glycolysis across kidney cell types, loss of cell-cell communication, and a partial reversal of vascular and metabolic aging features on SGLT2i treatment. We show new pathways, but we also establish a novel kidney model for aging researchers.

We observed an increase in interstitial fibrosis in aged kidneys. The main structural hallmark of kidney aging is nephrosclerosis, which is defined by glomerulosclerosis, tubular atrophy, interstitial fibrosis, and arteriosclerosis. Our histologic analyses revealed that aged killifish kidneys develop classic features of nephrosclerosis, closely mirroring those described in aging human kidneys and rodent models.^2,20^ Interestingly, we did not observe (global) glomerular hypertrophy during killifish aging: the mean diameter of glomeruli in aged kidneys stayed similar to that of young animals, but the overall distribution of various sizes increased significantly.

We found an increase in glomerular permeability in aged kidneys through leakage of molecules around 70 kDa (like albumin). Although albuminuria is not a prominent hallmark of human aging, it is frequently observed in aging rats and certain mouse strains, such as C57BL/6.^2^

We further established age-dependent changes in tubular function. Functional changes observed in mammalian kidneys include changes in tubular reabsorption and secretory capacities, leading to compromised metabolism and clearance of drugs,^20,21^ which can eventually lead to acute interstitial nephritis. We observed diverse age-associated changes in secretion of methotrexate via the MRP2 transporter, an ATP-binding cassette transporter. On the other hand, we were not able to observe differences in SGLT2 function. Isolated proximal tubules of Atlantic killifish (*Fundulus heteroclitus*) have been used as a model for pharmacodynamic studies.^22,23^ Atlantic killifish are caught in the wild, and therefore their age cannot be determined. Future studies in the African turquoise killifish will allow for more detailed analysis in tubular transport capacities in an age- and sex-specific manner (L. Kraus *et al*., 2024, unpublished data).

We found a significant decrease in microvascular density during the aging process. Vascular rarefaction not only affects vascular density but also EC integrity and pericyte-endothelial signaling.^3,24,25^ We observed a decline in CD31-positive staining and reduction of EC and perivascular cell counts in killifish aging. Consistent with other studies, we observed an ~45% decline in perivascular cell numbers with age, resembling the 30% to 50% pericyte loss seen in rodent models of fibrosis after ischemia.^26,27^

Peritubular capillary loss impairs oxygen delivery, disrupts metabolic homeostasis, and initiates fibrosis and inflammation.^26,28,29^ As such, microvascular rarefaction is increasingly recognized not merely as a consequence but as a driving factor in the progression of kidney fibrosis and age-related kidney functional decline.^30–32^ Pronounced microvascular rarefaction was a central finding in aged killifish kidneys, evidenced by loss of capillaries, total vessel volume, reduced vessel length, and decline of vessel branching. This is consistent with observations in aged mice, where cortical capillary rarefaction precedes glomerular damage,^33,34^ as well as human kidney transplant recipients, where amount of peritubular capillaries predicts renal function outcome.^35^

Single-nucleus transcriptomic analysis revealed that aging is associated with widespread downregulation of mitochondrial oxidative phosphorylation and tricarboxylic acid cycle pathways across all major kidney cell types. The shift from mitochondrial oxidative metabolism toward glycolysis observed in aged killifish kidneys mirrors metabolic inflexibility described in aging mammal kidney^36^ as well as other killifish organs during aging, like the liver.^37^ Concordantly, we observed an age-related loss of ATP-generating pathways and downregulation of fatty acid metabolism and amino acid metabolism gene sets, suggesting impaired metabolic adaptation. Loss of metabolic flexibility also underlies vascular and parenchymal deterioration during aging, in line with recent models reviewed by Augustin and Koh,^38^ in 2024.

Aged endothelial and perivascular cells exhibited significant loss of cell-cell adhesion molecules, leading to compromised vascular barrier integrity. We further observed an age-associated increase in inflammation markers. This matches a report suggesting that aging and chronic low-grade inflammation progressively destabilize endothelial junctions in other animal models, such as mice and zebrafish.^39^ Our findings demonstrate that structural deterioration of cell junctions can occur even in the absence of overt inflammation, emphasizing a direct aging effect and “inflammaging.”

Dapagliflozin treatment restored vascular density, endothelial communication pathways, and reduced albumin leakage in aged killifish kidneys. These effects are consistent with rodent studies showing that SGLT2i preserve endothelial function following kidney injury.^40^ Our study extends this observation to an aging context, suggesting that SGLT2i may be able to partially reprogram ECs. One possible explanation may be the observed upregulation of angiogenic factors, such as VEGF and ANGPTL4. These factors were only transcriptionally upregulated in SGLT2i-treated old kidneys. This observation is in line with in aging mice kidneys emphasizing the protective role of VEGF in vascular maintenance and chronic kidney disease prevention.^26,41–44^

We relied on pseudotime ordering that revealed an age-related decline in junctional and extracellular matrix gene expression, consistent with reduced endothelial communication detected by CellChat. SGLT2 inhibition decreased the proportion of damaged capillary cells and restored a more youthful pseudotime profile. The bimodal distribution observed under treatment suggests that SGLT2 inhibition maintains a subset of ECs in a stable, low pseudotime state while allowing continued turnover of others, thereby preserving microvascular integrity.

The resolution of age-dependent loss of metabolic flexibility observed in our study may suggest that SGLT2 inhibition also confers protective effects in the context of acute kidney injury, as previously reported in rodents and humans.^9,45,46^ We are currently investigating age-dependent acute kidney injury resolution and kidney regeneration in killifish as part of an ongoing study (T. Boewer *et al.*, 2025, unpublished data).

We also observed that the effects of SGLT2i on vasculature showed sex-dependent differences in vessel length and branching points. A possible explanation could be sex-dependent differences in the pharmacodynamics and pharmacokinetics of SGLT2i, also previously described by Miller *et al.*^47^ Despite clear structural and transcriptional improvements with SGLT2 inhibition, we did not observe a significant lifespan extension in treated killifish. This echoes observations from mammalian studies where the benefits of SGLT2 inhibition on vascular and metabolic health do not uniformly translate into lifespan gains, depending on timing and length of drug intake and sex-specific differences.^47^ The ideal dosing of drugs in a sex-specific manner and the translation of findings in killifish and mice to humans require further research.

Our study has limitations. The SGLT2i intervention cohort was relatively small, which may have limited the detection of subtle treatment effects on lifespan. In addition, the fish used in our experimental protocol showed a generally increased lifespan compared with the conventional cohort. This effect could be due to differences in water temperature^48^ and a difference in diet (see Supplementary Material – Diets), highlighting protein source and dietary regimen as drivers of longevity.^6,49^ Sex-specific data acquisition and analysis was not performed on histologic staining as well as tubular assays in this article. Where applicable, we refer readers to Supplementary Figure S2 for sex-specific comparisons. Recently, the killifish kidney has attracted attention as an immunologic organ; however, our study focused exclusively on the renal system and did not investigate hematopoietic or immune-related pathways that have been described previously.^50,51^

In summary, we characterized aging kidneys in a novel animal model of aging. We observed an increase in fibrosis and proteinuria and diminished tubular transport mechanisms in aged kidneys. Our study describes important hallmarks of aging^52,53^ in the kidneys of aging killifish. Mitochondrial dysfunction, including impaired ATP production, inflammation, and loss of metabolic flexibility, plays a central role in aging of all cell types. These changes are associated with altered intercellular communication between ECs, perivascular cells, and podocytes. SGLT2 inhibition not only preserves vascular networks but also reinstates “youthful” transcriptional programs, offering a strategy to mitigate age-associated kidney decline.

## Supplementary Material

1

2

3

figs2

figs6

figs4

figs3

figs1

figs5

figs7

Supplementary material is available online at www.kidney-international.org.

## Data Availability

The transcriptomic data supporting the findings of this study are openly available on publication in the Gene Expression Omnibus (GEO) under accession number GSE297623 (https://www.ncbi.nlm.nih.gov/geo/query/acc.cgi?acc=GSE297623). During peer review, the data were accessible to editors and reviewers using a secure token, which is available on request from the corresponding author. All raw and processed imaging data are available via our institutional OMERO server: https://omero-pub.mdibl.org/pubs/paulmann-et-al-2025

## Translational Statement

This study establishes the African turquoise killifish as a model for investigating kidney and vascular aging. We found that sodium-glucose linked transporter 2 (SGLT2) inhibition preserves microvascular integrity and reduces proteinuria. These results mirror established benefits observed in mammalian models and patients with chronic kidney disease, reinforcing the kidney-protective role of SGLT2 inhibitors. However, the killifish offers a unique opportunity for rapid, translational aging research. By using a naturally short-lived vertebrate with mammalian-like kidney aging, our model enables a rapid, preclinical assessment of vascular outcomes and identifies microvascular preservation as a potential mechanistic target for renoprotection.
