# Supplementary material for: Sodium-glucose co-transporter 2 inhibition improves age-dependent kidney microvascular rarefaction
Source: Kidney Int. Author manuscript; Available in PMC 2026 Apr 16. (PMC13085938; doi:10.1016/j.kint.2025.12.011)
Supplement: 1 [file NIHMS2154265-supplement-1.docx]

# **Fish diet and food composition**

### Killifeast® (Brine Shrimp Direct, USA)

Ingredients: Highly digestible marine fish hydrolysate, fresh frozen zooplankton (krill and mysis), yeast, soy, wheat gluten, wheat flour, egg, fish oil, lecithin, arctic copepod powder, casein, krill meal, spirulina, garlic, Paracoccus (natural source of astaxanthan), stabilized Vitamin C, carophyll, beta-carotene, vitamin-mineral premix, and beneficial bacteria. No artificial colors or preservatives added.

Proximate Analysis: Protein, 52.2%; Fat, 9.4%; Ash, 5.9%; Moisture, 9.1%; Fiber, 1.9%; Vitamin C, 2,000 ppm*; Astaxanthin, 400 ppm*. [Wasatch Laboratories, May 2018]

*
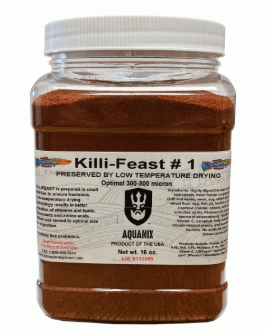
*

*Source:* [*https://www.brineshrimpdirect.com/pellets-and-wafers-sinking-and-floating/killifish-feast-plus/*](https://www.brineshrimpdirect.com/pellets-and-wafers-sinking-and-floating/killifish-feast-plus/)

### Repashy® Grub Pie Insectivore Gel Premix (Repashy® Speciality Pet Products, USA):

Ingredients: Black Soldier Fly Larvae Meal, Dried Seaweed Meal, Lecithin, Locust Bean Gum, Calcium Carbonate, Citric Acid, Taurine, Dried Kelp, Dried Watermelon, RoseHips, Hibiscus Flower, Marigold Flower, Paprika, Turmeric, Salt. Calcium Propionate, Sorbic Acid and Mixed Tocopherols (as preservatives). Magnesium Amino Acid Chelate, Zinc Methionine Hydroxy Analogue Chelate, Manganese Methionine Hydroxy Analogue Chelate, Copper Methionine Hydroxy Analogue Chelate. Vitamins: (Vitamin A Supplement, Vitamin D3 Supplement, L-Ascorbyl-Polyphosphate, Vitamin E Supplement, Niacin, Beta Carotene, d-Calcium Pantothenate, Riboflavin, Pyridoxine Hydrochloride, Thiamine Mononitrate, Folic Acid, Biotin, Vitamin B-12 Supplement, Menadione Sodium Bisulfite Complex).

GUARANTEED ANALYSIS: Crude Protein min. 40 %, Crude Fat min. 12%, Crude Fiber max. 1.5%, Moisture max. 10%, Ash max. 12%, Calcium min. 1.5%.


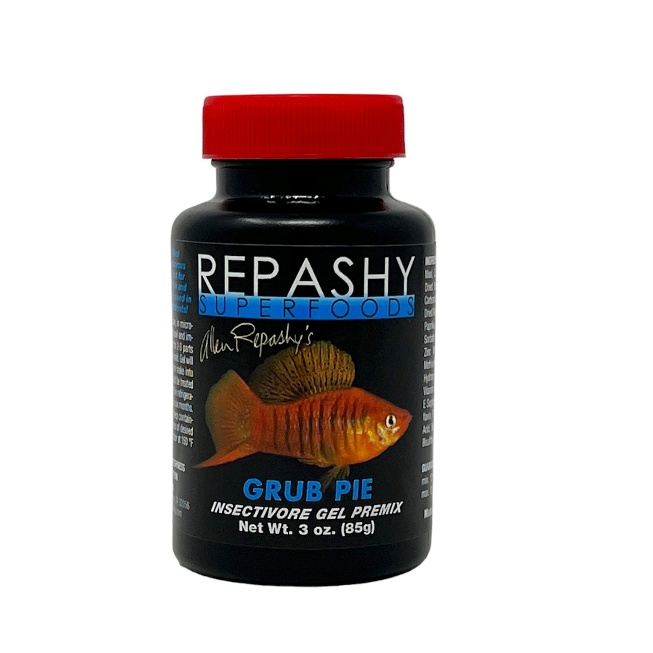


*Source: https://www.shop.repashy.com/products/repashy-grub-pie*

### Repashy® Redrum Carotenoid Gel Premix (Repashy® Speciality Pet Products, USA):

Ingredients: Krill Meal, Seaweed Meal, Locust Bean Gum, Pfaffia Rhodozyma Yeast, Paprika, Marigold Flower, Spirulina, Dried Watermelon, Rose Hips, Hibiscus Flower, Turmeric, Lecithin, Citric Acid, Calcium Propionate and Potassium Sorbate (as preservatives), Magnesium Amino Acid Chelate, Zinc Methionine Hydroxy Analogue Chelate, Manganese Methionine Hydroxy Analogue Chelate, Copper Methionine Hydroxy Analogue Chelate. Vitamins: (Vitamin A Supplement, Vitamin D Supplement, Calcium L-Ascorbyl-2-Monophosphate, Vitamin E Supplement, Niacin, Beta Carotene, d-Calcium Pantothenate, Riboflavin, Pyridoxine Hydrochloride, Thiamine Mononitrate, Folic Acid, Biotin, Vitamin B-12 Supplement, Menadione Sodium Bisulfite Complex).

GUARANTEED ANALYSIS: Crude Protein min. 30%, Crude Fat min. 15%, Crude Fiber max. 8%, Moisture max. 8%, Ash max. 9%.


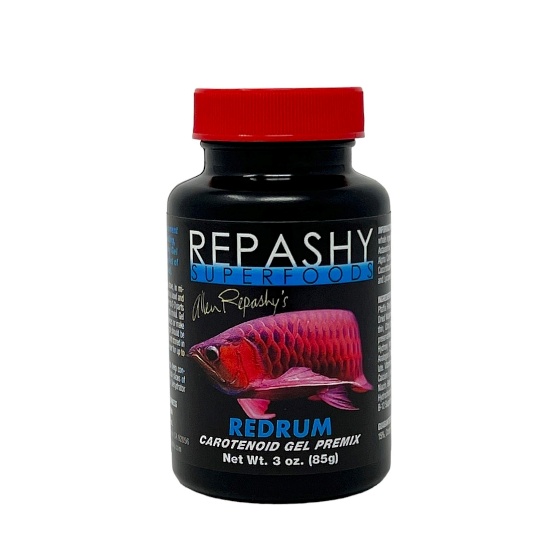


*Source:* [*https://www.shop.repashy.com/products/repashy-redrum*](https://www.shop.repashy.com/products/repashy-redrum)

*
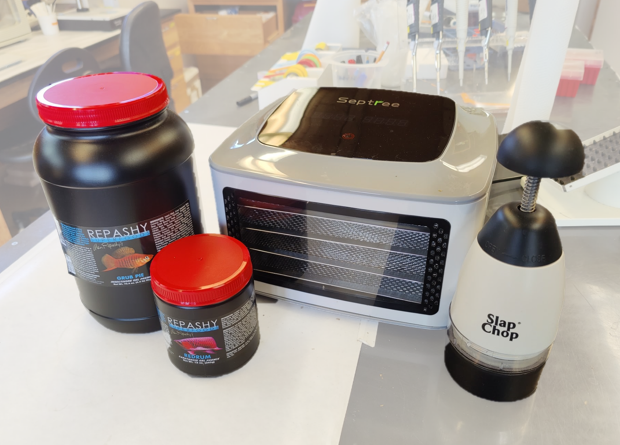
*

### Image of custom-diet fish food materials

- - Ingridients for Repashy mix (3 teaspoons *Grub* *Pie* and ¾ tsp *Redrum* per 200ml H_2_O )
  - food dehydrator (Septree^TM^, USA)
  - Slap^TM^Chop (USA) for chopping pellets

# **Comparison of Killifeast and Repashy Mix Diets**

| Parameter | Killifeast #1 | Repashy Mix |
| --- | --- | --- |
| Crude Protein | 52.2% | 35.3% |
| Crude Fat | 9.4% | 8.7% |
| Ash | 5.9% | 11.0% |
| Moisture | 9.1% | 8.0% |
| Crude Fiber | 1.9% | 3.5% |
| Primary Protein Sources | - Marine fish hydrolysate - Krill & mysis - Egg - Casein - Copepod powder | - Black Soldier Fly Larvae - Krill Meal (Redrum) - Plant-based (e.g., spirulina, seaweed, yeast) |
| Lipid Sources | - Fish oil - Lecithin | - Lecithin - Insect fat (larvae) - Krill oil (from Redrum) |
| Notable Additives & Extras | - Spirulina - Garlic - Probiotic bacteria - Astaxanthin (400 ppm) - Stabilized Vitamin C (2,000 ppm) | - Spirulina, paprika, turmeric, hibiscus, calendula - Phaffia yeast (astaxanthin) - Full vitamin/mineral premix - Preservatives (calcium propionate, potassium sorbate) |
| Color Enhancers / Antioxidants | - Astaxanthin (Paracoccus) - Carophyll - Beta-carotene | - Astaxanthin (Phaffia yeast) - Beta-carotene - Turmeric - Paprika |
| Probiotics / Functional Additives | Yes (beneficial bacteria included) | No live probiotics, but rich in plant-based bioactives |
| Preservatives | None (no artificial preservatives listed) | Calcium propionate, potassium sorbate |
