# Supplementary material for: Sodium-glucose co-transporter 2 inhibition improves age-dependent kidney microvascular rarefaction
Source: Kidney Int. Author manuscript; Available in PMC 2026 Apr 16. (PMC13085938; doi:10.1016/j.kint.2025.12.011)
Supplement: 2 [file NIHMS2154265-supplement-2.docx]

The ARRIVE reporting checklist

For checking that articles describing *in vivo* animal experiments can be understood and used by everyone

|  | Item Description | Location (or reason for not reporting) |
| --- | --- | --- |
| **Essential 10** |  |  |
| 1. Study Design |  |  |
| [1a. The groups being compared](https:/resources.equator-network.org/guidelines/arrive/items/groups-being-compared.html) | Groups being compared (young vs. old; treated vs. untreated) | Methods; "Animal Husbandry", "Histology" and throughout Results |
| [1b. The experimental unit](https:/resources.equator-network.org/guidelines/arrive/items/experimental-unit.html) | Experimental unit = individual fish | Methods; "Animal Husbandry" |
| 2. Sample Size |  |  |
| [2a. Number of Experimental Units](https:/resources.equator-network.org/guidelines/arrive/items/number-of-experimental-units.html) | Sample size per group clearly stated, total number of animals used for experiments n=74, total number of animals for lifespans n=1066 | Methods; “Statistical Analysis”; and in figure legends |
| [2b. Sample Size Justification](https:/resources.equator-network.org/guidelines/arrive/items/sample-size-justification.html) | No formal power calculation; sample size based on feasibility and previous studies. | Methods; “Statistical Analysis” |
| 3. Inclusion and Exclusion Criteria |  |  |
| [3a. Inclusion and exclusion criteria](https:/resources.equator-network.org/guidelines/arrive/items/inclusion-and-exclusion-criteria.html) | Inclusion: age, sex, health; no a priori exclusion criteria | Methods; “Animal Husbandry” |
| [3b. Exclusions and Attritions](https:/resources.equator-network.org/guidelines/arrive/items/exclusions-and-attritions.html) | Exclusions described (e.g., outliers in multiple replicates) | Methods; “Statistical Analysis” |
| [3c. Numbers analysed](https:/resources.equator-network.org/guidelines/arrive/items/numbers-analysed.html) | \|  \| \| --- \|  \| n values per analysis provided \| \| --- \| | Figure legends |
| 4. Randomisation |  |  |
| [4a. Randomisation Use](https:/resources.equator-network.org/guidelines/arrive/items/randomisation.html) | Randomisation was used to allocate animals to control and intervention groups. Animals were randomly assigned by selecting half of the cohort for the treatment group and the other half for the control group. All animals were age-matched and had equal gender distribution at the start of the experiment. | Methods; “Animal Husbandry” |
| [4b. Confounders](https:/resources.equator-network.org/guidelines/arrive/items/confounders.html) | Confounders (e.g., sex, housing) discussed in Results/Discussion. | Discussion; Supplementary Figures |
| [5. Blinding/Masking](https:/resources.equator-network.org/guidelines/arrive/items/blinding.html) | Blinding not explicitly performed | Not reported |
| 6. Outcome Measures |  |  |
| [6a. Outcome Measures](https:/resources.equator-network.org/guidelines/arrive/items/outcome-measures.html) | Defined outcomes: lifespan, histological markers (e.g. glomerulosklerosis, tubulosclerosis, arteriosclerosis, Sirius red-positive area), CD31-positive area, albumin-positive area, vascular parameters (vessel density, length, branchpoints, for vessel network as well as individual vessels), transcriptomics (cell counts, gene counts) | Methods; “Functional Assays and Quantification” |
| [6b. Primary Outcome Measure](https:/resources.equator-network.org/guidelines/arrive/items/primary-outcome-measure.html) | Primary outcome: vascular rarefaction | Abstract; Results |
| 7. Statistical Methods |  |  |
| [7a. Statistical Methods used for each Analysis](https:/resources.equator-network.org/guidelines/arrive/items/statistical-methods-analysis-methods.html) | Statistical methods listed (ANOVA, t-test, etc.) in GraphPad Prism | Methods; “Statistical Analysis” |
| [7b. Statistical Assumptions](https:/resources.equator-network.org/guidelines/arrive/items/statistical-methods-assumptions.html) | Normality testing via Shapiro-Wilk | Methods; “Statistical Analysis” |
| 8. Experimental Animals |  |  |
| [8a. Species-appropriate Details](https:/resources.equator-network.org/guidelines/arrive/items/experimental-animals-species-appropriate-details.html) | Species/strain: N. furzeri, GRZ strain; both sexes, ages defined | Methods; “Animal Husbandry” |
| [8b. Further Information](https:/resources.equator-network.org/guidelines/arrive/items/experimental-animals-further-information.html) | All Wildtype animals | Methods; “Animal Husbandry” |
| 9. Experimental Procedures |  |  |
| [9a. What was done](https:/resources.equator-network.org/guidelines/arrive/items/experimental-procedures-what-was-done.html) | All procedures, timepoints, and tools described in Supplementary Methods | Methods sections throughout |
| [9b. When and how often procedures were conducted](https:/resources.equator-network.org/guidelines/arrive/items/experimental-procedures-when-and-how-often.html) | Descriptive statistics and graphs shown. Controls and Interventional Analysis done in parallel, experiments were repeated on average 2-5 times. | Figure legends; Results |
| [9c. Where procedures were conducted](https:/resources.equator-network.org/guidelines/arrive/items/experimental-procedures-where.html) | All experiments were conducted at the MDIBL. | Methods |
| [9d. Why procedures were done](https:/resources.equator-network.org/guidelines/arrive/items/experimental-procedures-why.html) | All procedures, timepoints, and tools described in Supplementary Methods | Methods sections throughout |
| 10. Results |  |  |
| [10a. Summary/Descriptive Statistics per group](https:/resources.equator-network.org/guidelines/arrive/items/results-summary-per-group.html) | Descriptive statistics and graphs shown throughout | Figure legends; Results |
| **Recommended Set** |  |  |
| [11. Abstract](https:/resources.equator-network.org/guidelines/arrive/items/abstract.html) | Provide an accurate summary of the research objectives, animal species, strain and sex, key methods, principal findings, and study conclusions. | See Abstract |
| 12. Background |  |  |
| [12a. Rationale](https:/resources.equator-network.org/guidelines/arrive/items/rationale.html) | Include sufficient scientific background to understand the rationale and context for the study, and explain the experimental approach. | Introduction |
| [12b. Species and model](https:/resources.equator-network.org/guidelines/arrive/items/species-and-model.html) | Explain how the animal species and model used address the scientific objectives and, where appropriate, the relevance to human biology. | Introduction, Results, Discussion |
| [13. Objectives](https:/resources.equator-network.org/guidelines/arrive/items/objectives.html) | Clearly describe the research question, research objectives and, where appropriate, specific hypotheses being tested. | Introduction |
| [14. Ethical statement](https:/resources.equator-network.org/guidelines/arrive/items/ethical-statement.html) | Provide the name of the ethical review committee or equivalent that has approved the use of animals in this study and any relevant licence or protocol numbers (if applicable). If ethical approval was not sought or granted, provide a justification. | Ethical approval described in Methods |
| [15. Housing and husbandry](https:/resources.equator-network.org/guidelines/arrive/items/housing-husbandry.html) | \| Housing at 27°C, light/dark cycle, etc. \| \| --- \|  \|  \| \| --- \| | Methods; “Animal Husbandry” |
| 16. Animal Care and Monitoring |  |  |
| [16a. Reducing pain, suffering, and distress](https:/resources.equator-network.org/guidelines/arrive/items/reducing-pain-suffering-distress.html) | Pain/distress minimized via MS-222 euthanasia | Methods |
| [16b. Adverse events](https:/resources.equator-network.org/guidelines/arrive/items/adverse-events.html) | Report any expected or unexpected adverse events. |  |
| [16c. Humane endpoints](https:/resources.equator-network.org/guidelines/arrive/items/humane-endpoints.html) | Humane endpoints were defined as difficulty swimming, corkscrewing, spinal deformities etc. Only applicable for lifespan analysis | Methods |
| 17. Interpretation/ scientific implications |  |  |
| [17a. Interpretation/scientific implications](https:/resources.equator-network.org/guidelines/arrive/items/interpretation-implications.html) | Interpretation consistent with objectives; limitations discussed | Discussion |
| [17b. Limitations](https:/resources.equator-network.org/guidelines/arrive/items/limitations.html) | Comment on the study limitations, including potential sources of bias, limitations of the animal model, and imprecision associated with the results. | Discussion |
| [18. Generalisability/translation](https:/resources.equator-network.org/guidelines/arrive/items/generalisability.html) | Generalizability to mammalian aging discussed | Discussion |
| [19. Protocol registration](https:/resources.equator-network.org/guidelines/arrive/items/protocol-registration.html) | No explicit protocol registration |  |
| [20. Data Access](https:/resources.equator-network.org/guidelines/arrive/items/data-access.html) | Data availability (snRNA-seq section) in GEO database | Methods |
| 21. Declaration of interests |  |  |
| [21a. Conflicts of interests](https:/resources.equator-network.org/guidelines/arrive/items/conflicts-of-interests.html) | Declare any potential conflicts of interest, including financial and nonfinancial. If none exist, this should be stated. | See Disclosures |
| [21b. Funding](https:/resources.equator-network.org/guidelines/arrive/items/funding.html) | List all funding sources (including grant identifier) and the role of the funder(s) in the design, analysis, and reporting of the study. | See Disclosures and Acknowledgements |
