# Supplementary figures and images for: Sodium-glucose co-transporter 2 inhibition improves age-dependent kidney microvascular rarefaction
Source: Kidney Int. Author manuscript; Available in PMC 2026 Apr 16. (PMC13085938; doi:10.1016/j.kint.2025.12.011)

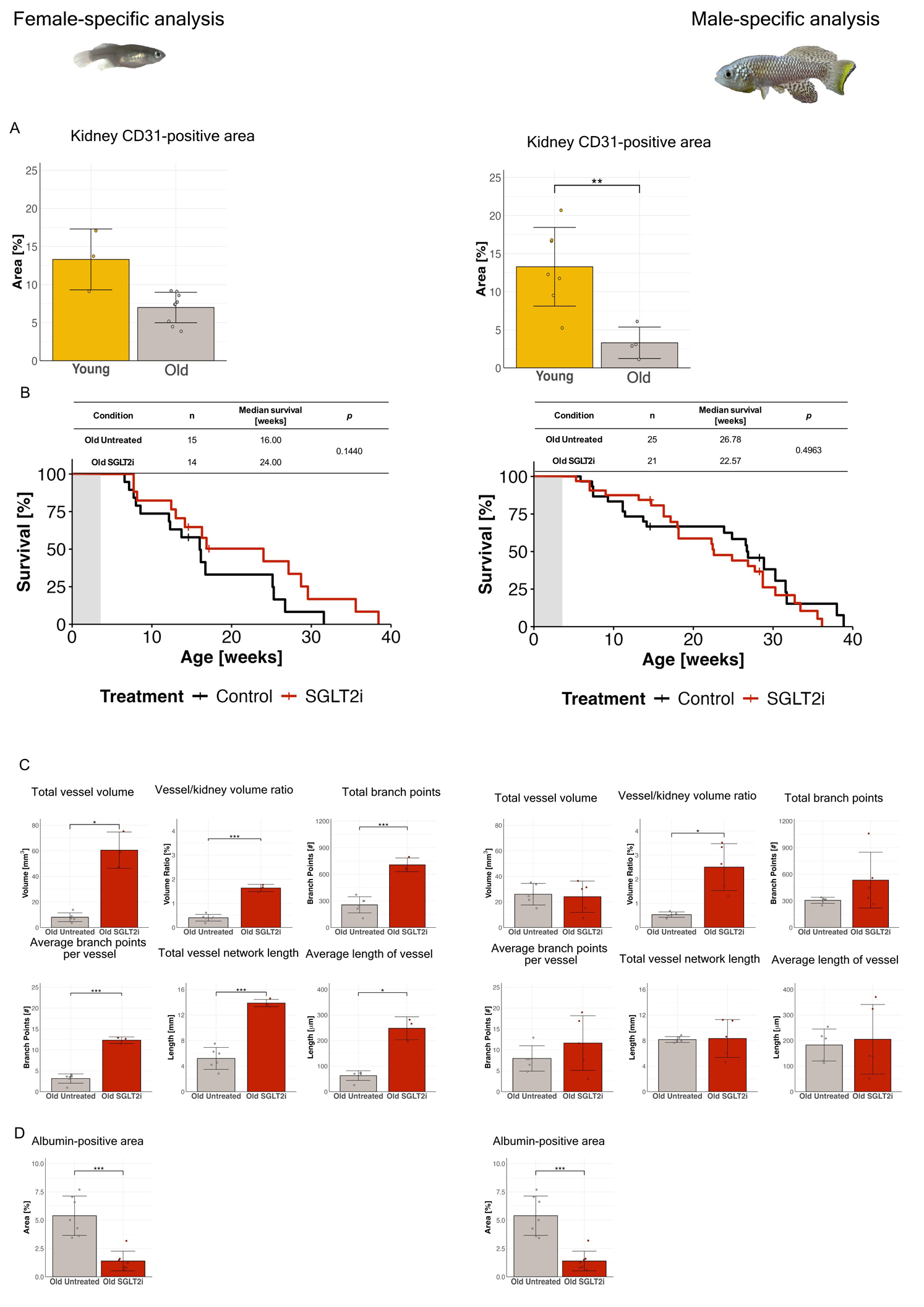

Supplement: figs2 [file NIHMS2154265-supplement-figs2.jpg]

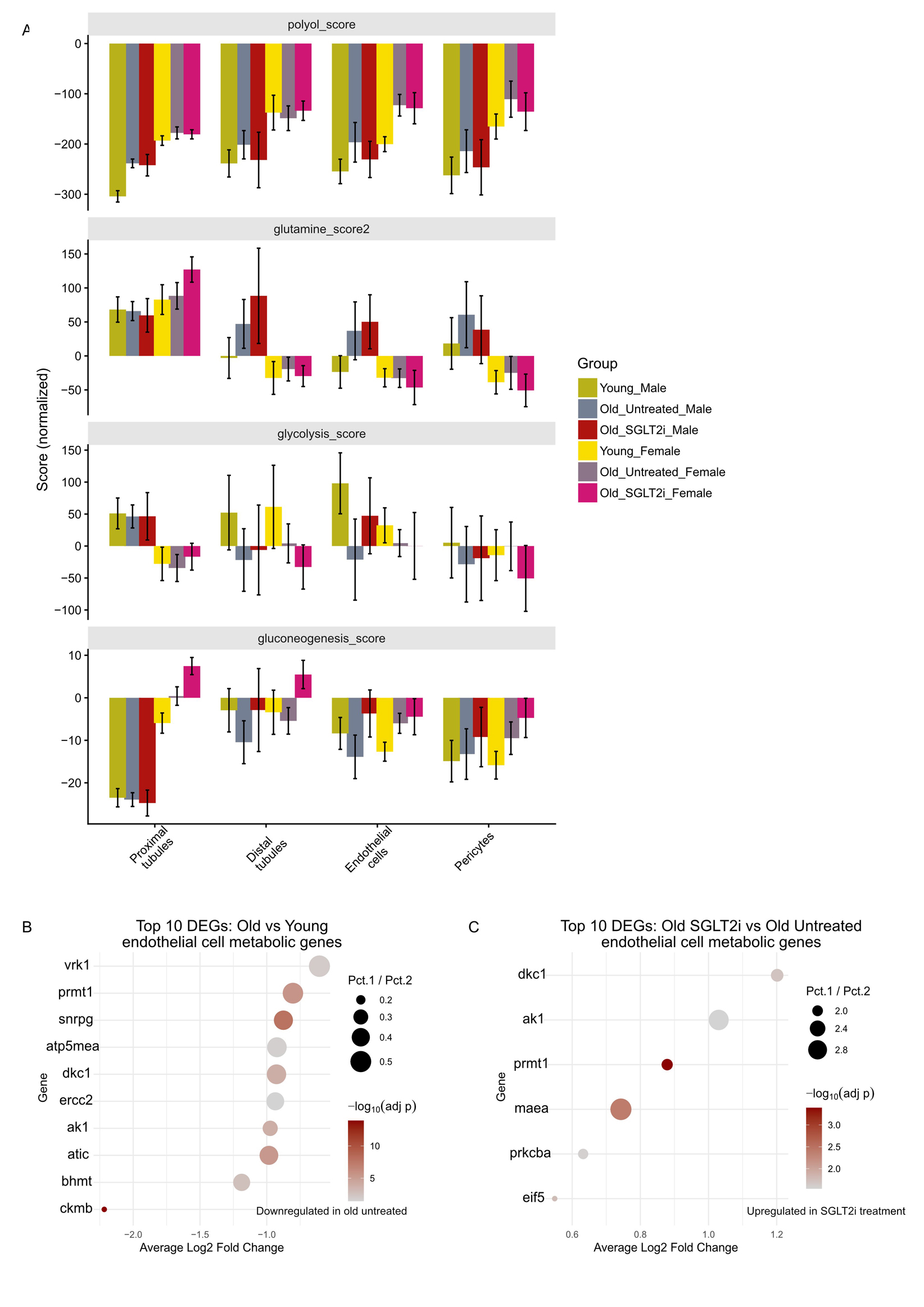

Supplement: figs6 [file NIHMS2154265-supplement-figs6.jpg]

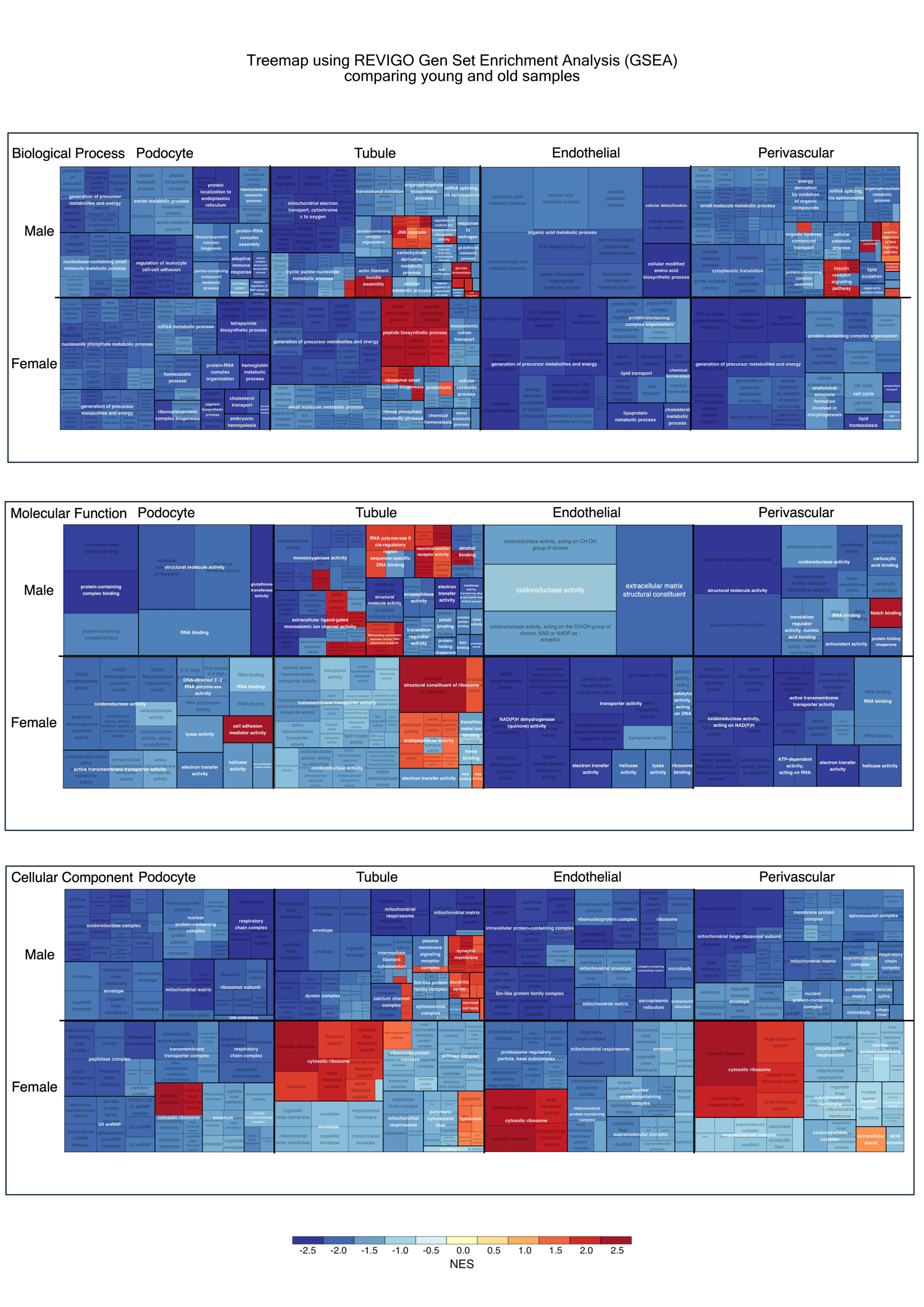

Supplement: figs4 [file NIHMS2154265-supplement-figs4.jpg]

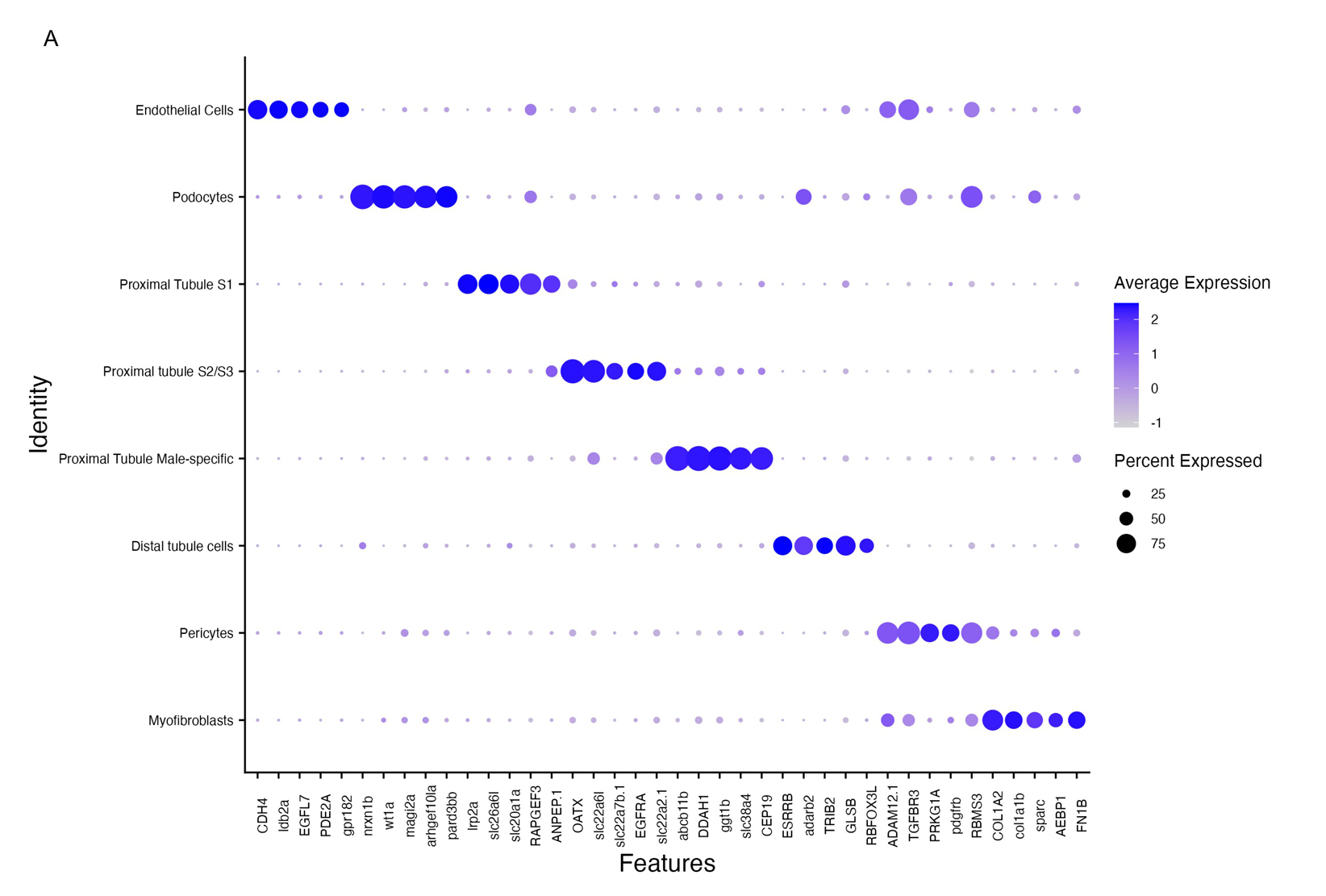

Supplement: figs3 [file NIHMS2154265-supplement-figs3.jpg]

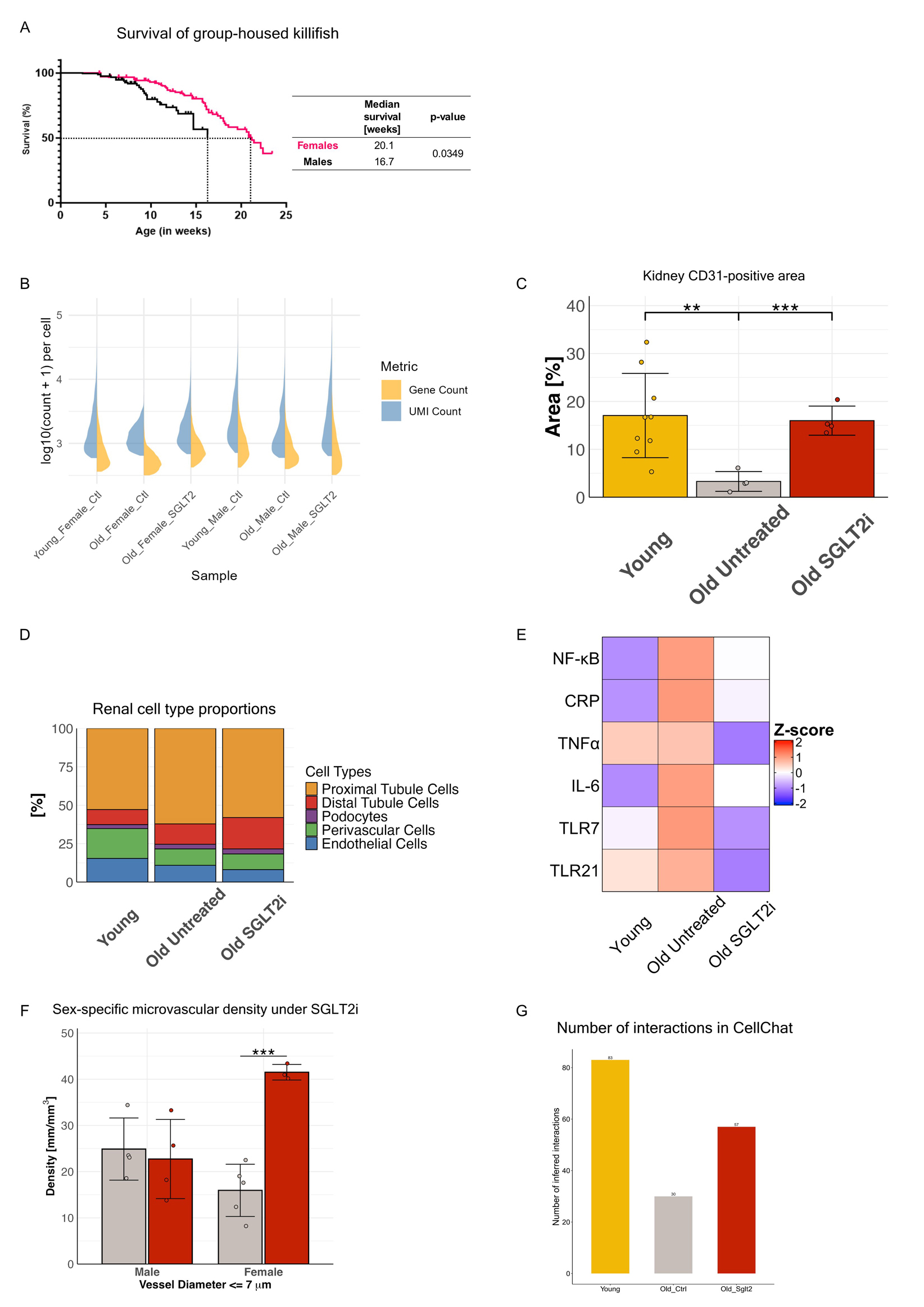

Supplement: figs1 [file NIHMS2154265-supplement-figs1.jpg]

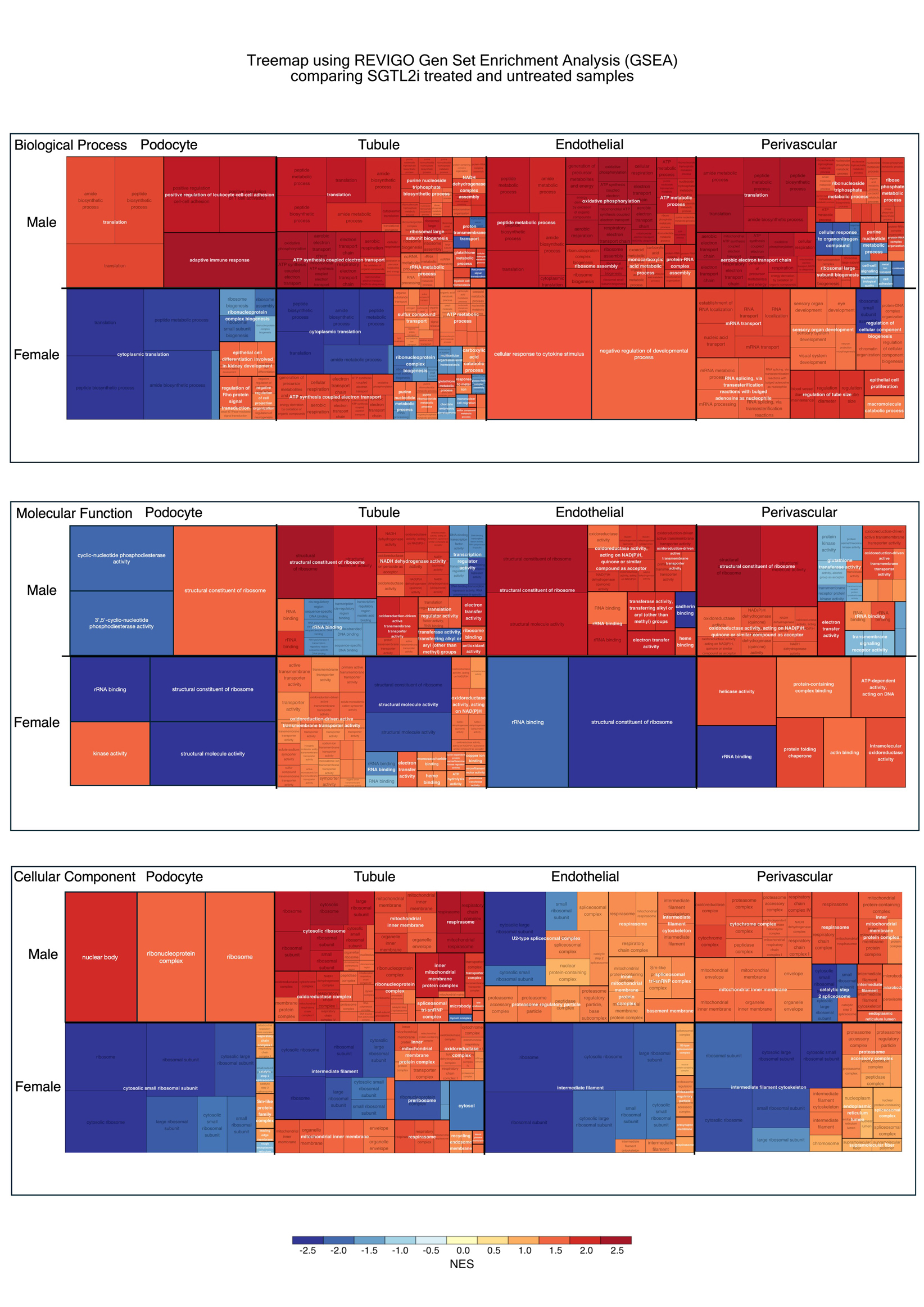

Supplement: figs5 [file NIHMS2154265-supplement-figs5.jpg]

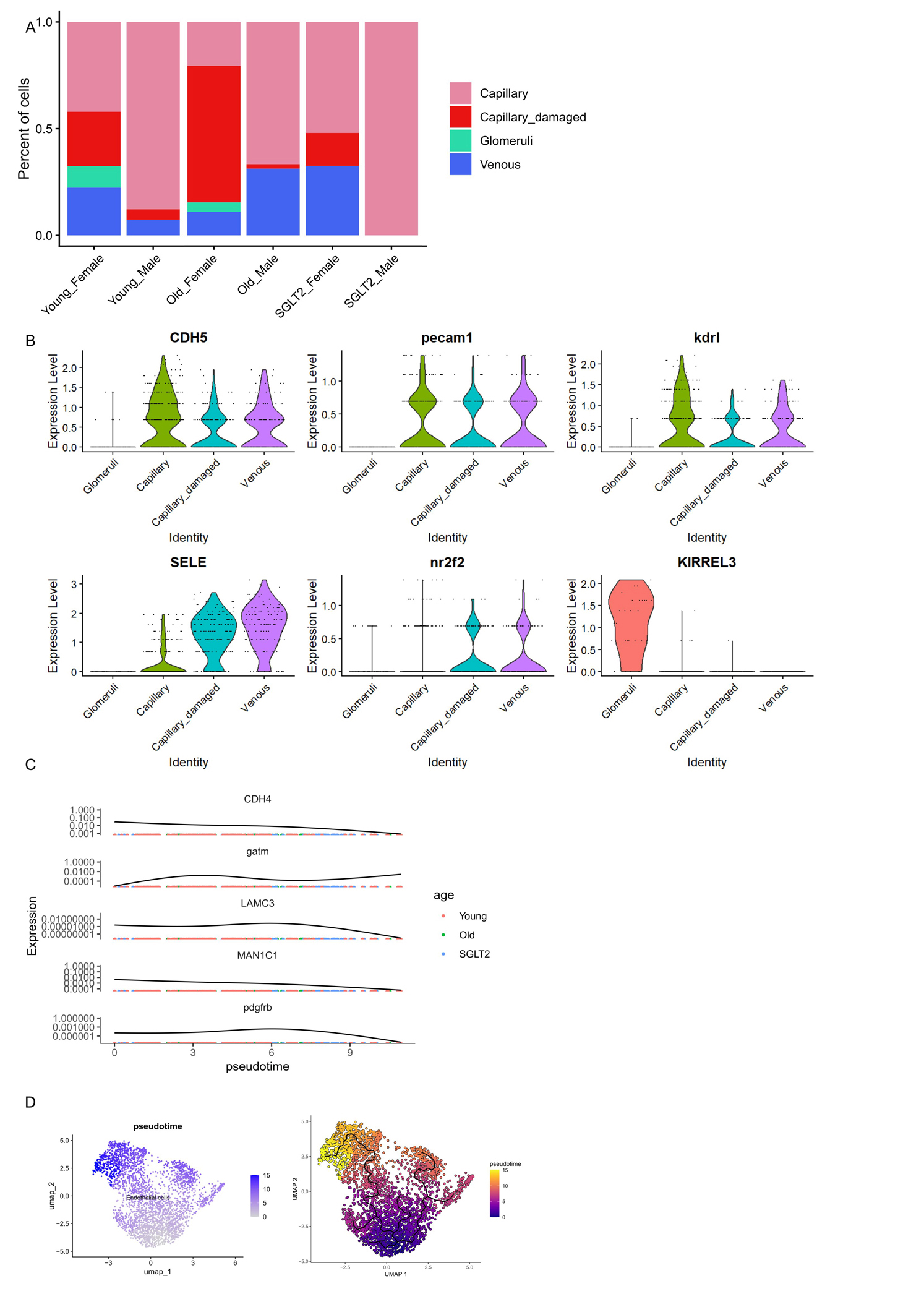

Supplement: figs7 [file NIHMS2154265-supplement-figs7.jpg]
